# Characterization of EGFR-reprogrammable temozolomide-resistant cells in a model of glioblastoma

**DOI:** 10.1038/s41420-022-01230-y

**Published:** 2022-10-31

**Authors:** Lingli Gong, Ying Yin, Cheng Chen, Quan Wan, Die Xia, Mei Wang, Zhening Pu, Bo Zhang, Jian Zou

**Affiliations:** 1 Department of Laboratory Medicine, The Affiliated Wuxi People’s Hospital of Nanjing Medical University, Wuxi, 214023 China; 2 Center of Clinical Research, The Affiliated Wuxi People’s Hospital of Nanjing Medical University, Wuxi, 214023 China; 3 Department of Neurosurgery, The Affiliated Wuxi Second Hospital of Nanjing Medical University, Wuxi, Jiangsu 214002 China

**Keywords:** CNS cancer, Cancer models

## Abstract

Temozolomide (TMZ) resistance is a major clinical challenge for glioblastoma (GBM). O^6^-methylguanine-DNA methyltransferase (MGMT) mediated DNA damage repair is a key mechanism for TMZ resistance. However, MGMT-null GBM patients remain resistant to TMZ, and the process for resistance evolution is largely unknown. Here, we developed an acquired TMZ resistant xenograft model using serial implantation of MGMT-hypermethylated U87 cells, allowing the extraction of stable, TMZ resistant (TMZ-R) tumors and primary cells. The derived tumors and cells exhibited stable multidrug resistance both in vitro and in vivo. Functional experiments, as well as single-cell RNA sequencing (scRNA-seq), indicated that TMZ treatment induced cellular heterogeneity including quiescent cancer stem cells (CSCs) in TMZ-R tumors. A subset of these were labeled by *NES*^+^*/SOX2*^+^*/CADM1*^+^ and demonstrated significant advantages for drug resistance. Further study revealed that Epidermal Growth Factor Receptor (EGFR) deficiency and diminished downstream signaling may confer this triple positive CSCs subgroup’s quiescent phenotypes and chemoresistance. Continuous EGF treatment improved the chemosensitivity of TMZ-R cells both in vitro and in vivo, mechanically reversing cell cycle arrest and reduced drug uptake. Further, EGF treatment of TMZ-R tumors favorably normalized the response to TMZ in combination therapy. Here, we characterize a unique subgroup of CSCs in MGMT-null experimental glioblastoma, identifying EGF + TMZ therapy as a potential strategy to overcome cellular quiescence and TMZ resistance, likely endowed by deficient EGFR signaling.

## Introduction

Glioblastoma (GBM), the most malignant human brain tumor, is characterized by resistance to anti-cancer therapy [1]. Temozolomide (TMZ), an oral alkylating agent, is one of the first-line drugs commonly used in chemotherapy of GBM [2, 3]. The cytotoxicity of TMZ is commonly attributed to O6-methylguanine-induced DNA damage, which in turn induces DNA crosslinking, resulting in tumor cell death [4]. The lack of response to TMZ treatment arises from the intrinsic or acquired resistance of tumors to recurrent drug exposure. The DNA repair enzyme, O^6^-methylguanine methyltransferase (MGMT)- mediated demethylation at the O^6^ site is a key pathway that has been implicated in intrinsic TMZ resistance [5]. In gliomas, the methylation level of MGMT is also closely related to the efficacy of TMZ, where patients with hypomethylation of the MGMT promoter displaying an improved response to TMZ and survival outcomes [6]. The expression of MGMT is negatively correlated with promoter methylation status and nearly half of GBM patients experience MGMT silencing due to hypermethylation of the promoter [7, 8]. While most of them eventually develop tumor progression and acquired resistance to TMZ [9], the available evidence indicates that both MGMT promoter methylation status and MGMT protein remain relatively stable during GBM progress and TMZ treatment [6, 10]. Given that the acquired resistance to TMZ in GBM is not likely to be associated with re-expression of MGMT [11], MGMT-independent mechanisms probably exist in the acquired resistance of MGMT-null GBM patients, which remain largely unknown.

Establishing a TMZ-resistance model is an important aim toward understanding the mechanisms implicated in TMZ-resistant GBM. Most TMZ-resistance cells are induced gradually by increasing drug concentration in vitro. Shortcomings exist in these in vitro models, including a long induction time, a largely different resistance profile (compared to in vivo resistance), and difficulty in long-term maintenance of drug resistance. A sunitinib-resistant subcutaneous model derived from renal cell carcinoma cell partially compensates for the deficiencies of the in vitro model [12]. Kitange developed an in vivo GBM intracranial model of TMZ resistance using short-term cultured patient-derived glioma cells. The TMZ resistance was induced by continuous high-dose TMZ therapy through in vivo continuous passage [13], highlighting a promising solution for exploring the underlying mechanisms of chemoresistance. However, few in vivo models of GBM-acquired chemoresistance have been developed to date.

Here, we produced an acquired TMZ-resistant (TMZ-R) model by performing three cycles of TMZ treatment along with in vivo tumor passage using U87 cells, a MGMT-null GBM cell line. The derived tumors and cells exhibited stable multidrug resistance both in vitro and in vivo. Functional experiments, as well as single-cell RNA sequencing (scRNA-seq), further indicated that TMZ treatment resulted in cellular heterogeneity which induced quiescent cancer stem cells (CSCs), labeled by *NES*^+^*/SOX2*^+^*/CADM1*^+^ and acting as a major contributor of TMZ resistance. This study also suggests that EGFR deficiency confers this CSCs subgroup’s cell cycle arrest and chemoresistance. Moreover, we provide evidence that EGF is a favorable solution to overcome TMZ chemoresistance, restoring the therapeutic response.

## Materials and methods

### Cells lines and culture conditions

The human GBM cell line U87 was purchased from the Chinese Academy of Science (Shanghai, China) and was verified using short tandem repeat assays by GENEWIZ (Suzhou, China). All cells were grown in DMEM (Hyclon, Logan, UT, USA) supplemented with 10% fetal bovine serum (FBS; Biological Industries, Beit-Haemek, Israel), 1% penicillin and streptomycin (P/S; Hyclon). To initiate stem-like induction, the cells derived from U87 xenografts were cultured in a defined serum-free neural stem cell (NSC) medium containing 20 ng/ml of basic fibroblast growth factor (bFGF, Peprotech, Rocky Hill, NJ, USA), 20 ng/ml of epidermal growth factor (EGF, Peprotech), N2 and B27 (Invitrogen). TMZ, Doxorubicin (DOX), Etoposide (Eto), Cis-platinum (CIS), and 5-Fluorouracil (5-Fu) were acquired from Selleck Chemicals.

### TMZ resistant GBM xenografts

**Fig. 1:**
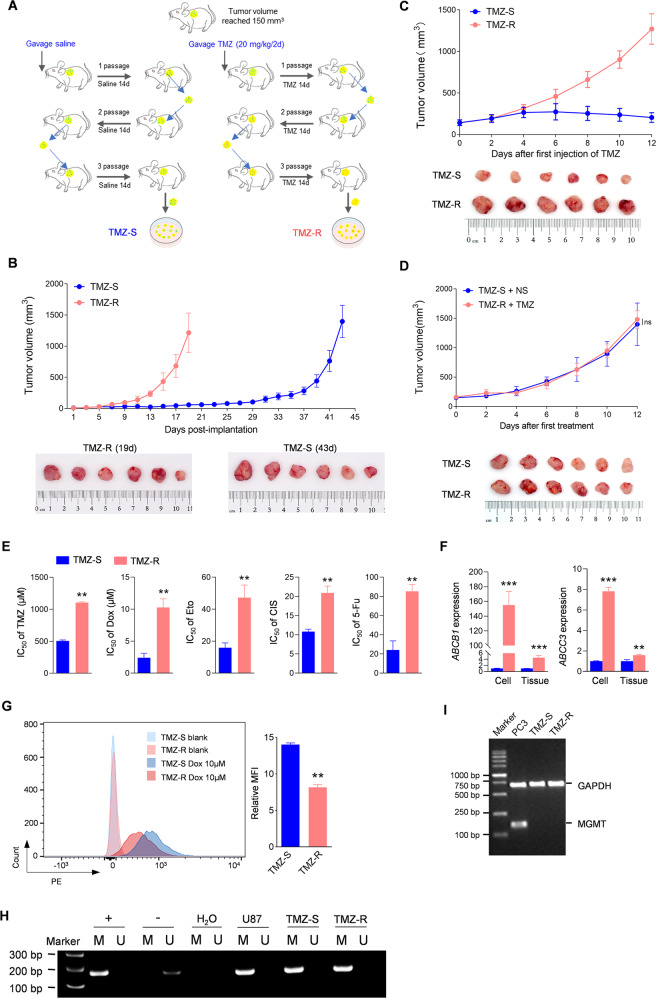
Construction and confirmation of an acquired TMZ-resistant GBM xenograft model. **A** Schematic process of the acquired TMZ-resistant GBM tumors and cells. **B** Tumor growth assay of cells derived from indicated xenografts. The lower panel showing the tumors collected at the final day. **C** Tumor growth assay of indicated xenografts after the first TMZ injection. Mice were subcutaneously injected with indicated cells and subsequently received intragastric administration of TMZ (20 mg/kg) every two days after the tumors reached 100 mm^3^. Tumor volume was monitored as indicated the day from the 1st TMZ injection. Xenografts were collected on the 12nd day post TMZ-treatment. **D** Tumor growth assay of xenografts received the indicated treatment. Mice were subcutaneously injected with indicated cells and subsequently received intragastric administration of normal saline (NS) or TMZ (20 mg/kg) every two days after the tumors reached 100 mm^3^. Tumor volume was monitored at the indicated day from the 1st treatment. Tumors were collected on the 12nd day post TMZ-treatment. **E** IC50 assay indicating different drug response between TMZ-S and TMZ-R cells (*n* = 3, **p < 0.01). **F** qRT–PCR assay measuring the expression of *ABCB1* and *ABCC3* mRNA in the indicated cells and tumors (*n* = 3, **p < 0.01). **G** Flow cytometry (FC) assay (left) and the statistical analysis of mean fluorescence intensity (MFI; right) showing the uptake of DOX by the indicated cells, the shift in fluorescence intensity of a population of cells (*n* = 3, **p < 0.01). **H** Analysis of MGMT promoter methylation in U87 cells and derived indicated tumors by methylation-specific PCR (MSP). M and U indicating methylated (M) and unmethylated (U) status of the promoter respectively; + and − representing the bisulfite-converted methylated and unmethylated DNA respectively. **I** RT-PCR measurement showing the expression of MGMT in indicated cells. PC-3 cells served as a MGMT-positive control.

Four weeks old male BALB/c nude mice were purchased from the Shanghai Animal Center, Chinese Academy of Sciences and maintained under specific pathogen-free conditions at Wuxi People’s Hospital. As these experiments were exploratory, there was no estimation to base the effective sample size; therefore, we based our animal studies using sample size=6. All mice were assigned randomly. To initiate tumors, 5 × 10^6^ of U87 cells in 100 μl of DMEM: Matrigel (8:1, v/v; BD Biosciences, Franklin Lakes, NJ) were injected subcutaneously into the flank of each nude mouse, and tumors were allowed to develop until they reached a volume of 100 to 150 mm^3^. Mice with lateral abdominal tumors were randomly divided into two groups, which were given saline and Temozolomide (TMZ, 20 mg/kg; Selleck, Houston, TX) once every other day for 14 days (Fig. 1A). The xenografts derived from the first generation of mice were sheared into small pieces of 1 mm^3^, and the tissue lysates were re-injected into the flank of naïve nude mice, who received TMZ treatment in the same manner. The stable TMZ drug-resistant strain was obtained after continuous passage for three cycles, as indicated. All animal care and handling procedures were performed in accordance with the National Institutes of Health’s Guide for the Care and Use of Laboratory Animals. All animal experiments were approved by the Institutional Review Board of Nanjing Medical University (No. (2020)353). All animal experiments were performed by two technicians blinded to the treatment condition of the mice. No mice were excluded from scoring.

### Cell growth inhibition assay

Cytotoxicity studies were performed in 96-well plates and optimal seeding densities for each cell line were determined to ensure exponential growth during the assay. Cells were cultured with medium containing gradient concentration of drugs for 48 h. Cell viability was determined by Cell Counting Kit-8 (CCK-8; Bimake, Houston, TX, USA). Optical density (OD) at 450 nM was measured using a microplate reader (Thermo, Waltham, MA, USA). The percentage of cell survival at each concentration was calculated by the formula: (OD_treated_/OD_untreated_) × 100. The IC_50_ value represented the drug concentration that reduced cell growth to 50%.

### Cell growth assay and colony formation assay

For cell growth assays, a total of 1000 cells were seeded into 96-well plates and monitored by Cell Counting Kit-8 according to the manufacture’s protocol at the indicated time points. For colony formation assays, 1000 cells were seeded into six-well plates and maintained in a complete medium for 14 days. The colonies were fixed with 4% paraformaldehyde (PFA) (Sangon, Shanghai, China) and stained with 0.1% crystal violet (Sangon, China), the number of colonies was counted using an inverted microscope.

### Cell cycle assay

Cells were cultured for 24 h followed by synchronization with serum deprivation for 24 h. After culture in normal medium for another 24 h, cells were harvested and fixed with 70% ethanol at −20 °C for 24 h. The derived cells were suspended in PBS containing 100 ng/ml of RNaseA (Boehringer Mannheim, Indianapolis, IN) and 50 μg/ml of propidium iodide (PI, Sigma), incubated for 1 h at RT in dark. The cell cycle fractions were measured by FACS Canto II (BD, Mountain View, CA). The percentages of cells in G1, S, and G2 phases were calculated using ModFit LT software (RRID:SCR_016106, BD).

### EdU labeling assay

EdU labeling assay was performed to examine the rate of DNA replication. In brief, cells were cultured in six-well plate for 24 h followed by an incubation of with 10 μM EdU (KeyGen Biotech, China) in normal culture conditions for 3 h. Cells were harvested and fixed with 4 % paraformaldehyde (PFA). The staining procedure was performed according to the manufacturer’s instructions of the Click-iT^TM^ EdU Alexa Fluor^TM^ 647 Flow cytometry Assay kit (Thermo). The EdU-positive cells were measured by flow cytometer (BD, FACS Canto II).

### Migration assay

2 × 10^4^ cells in 200 μL serum-free medium were placed onto the top transwell chamber (8-μm pore size, BD Biosciences) of each insert. After 24 h of incubation at 37°C, cells adhering to the lower membrane were stained with 0.1% crystal violet in 20% methanol, imaged, and counted using an EVOS FL microscope (Life technology, Gaithersburg, MD, USA).

### 3D spheroids assay

Spheroid formation and growth were analyzed as described previously [14]. Briefly, 400 cells were seeded in spheroid microplate (Corning Inc., NY, USA) according to a 3D-spheroids formation and growth protocol. The spheroids were cultured with GBO medium with a minor modification (without insulin) according to a previously published report [15], changing half of the medium every 2 days. The growth of the 3D-Spheroid cultured cells was monitored by a microscope with a real-time camera (EVOS® FL Auto Imaging System, Life Technologies, Carlsbad, CA, USA). For sphere growth assay, photographs of tumor spheres were taken at the indicated time points and sphere diameter was measured to reflect sphere growth.

### Reverse-transcription semi-quantitative PCR (RT-PCR) and quantitative PCR (RT-qPCR)

Total RNA was isolated using Trizol reagent (Thermo) according to the manufacturer’s instructions. HiFiScript cDNA synthesis Kit (CoWin Bioscience, China) was used according to the manufacturer’s protocol. Quantitative PCR (qPCR) analyses were conducted to quantify mRNA relative expression using Real SYBR Mixture (CoWin Bioscience), with GAPDH as an internal control. For semi-quantitative PCR of MGMT expression, total RNA was isolated and first-strand cDNA was generated according to the manufacturer’s instructions. PCR was performed with PrimeSTAR Max DNA Polymerase (Takara). The PCR products were separated by agarose gel electrophoresis, and scanned with a Gel imager (Tanon, China). The primers are shown in Supplementary Tables 1 and 2.

### Methylation-specific PCR

The MGMT promoter methylation status was determined by methylation-specific PCR (MSP). Briefly, 2 μg of DNA were subjected to bisulfite treatment using the EpiArt® DNA Methylation Bisulfite Kit (Vazyme; EM101). DNA was cleaned up following the manufacturer’s instructions and quantified. 30 ng of DNA per sample were PCR-amplified with the EpiArt^TM^ HS Taq DNA Polymerase (Vazyme, EM201) and specific primers to detect methylated and unmethylated MGMT promoter (Supplementary Table 2). The PCR amplification protocol was as follows: 95 °C for 5 min, denature at 95 °C for 30 s, anneal at 60 °C for 30 s, extension at 70 °C for 30 s for 40 cycles, followed by a 5 min final extension.

### Single-cell RNA-sequencing and data analysis

Single-cell RNA sequencing was performed using 10× Chromium single-cell platform (10X Genomics). Briefly, cells were washed with a phosphate buffer solution containing 0.04% weight/volume bovine serum albumin (BSA, Sangon). Cells were counted using Countess^®^II Automated Cell Counter and the concentration was adjusted to 1 × 10^6^ /ml. The cDNA libraries were constructed using the 10× Chromium TM Single cell 3’ Library Kit according to the manufacturer’s original protocol available on the 10x Genomics website. Cell Ranger 1.3 (http://10xgenomics.com) was used to process Chromium single cell 3’ RNA-seq output. The R package ‘Seurat’ (Version 3.2.3) was used to the initial clustering and Loupe Cell Browser 3.1.0 was used to view the clustering. The R package “SingleR” (Version 1.0.1) was utilized to identify the predominant cell types. Single-cell pseudotime trajectories were constructed with the R package Monocle (Version 2.1.0) [16]. Gene set variation analysis (GSVA) was also performed by the R package. GSVA (Version 1.32.0) was used to estimate the activity for diverse signatures and pathways [17]. GSVA scores for indicated signatures were calculated using predefined gene sets obtained from the MSigDB (Molecular Signatures Database) (http://www.gsea-msigdb.org/gsea/downloads.jsp).

### Multiplex immunohistochemistry/immunofluorescence (mIHC/IF)

mIHC/IF was performed using an Opal 7-color Manual IHC Kit (PerkinElmer, USA), as previously described in other studies [18, 19]. Tissue sections (4 µm thick) were labeled with primary antibodies against EGFR, Nestin, Sox2, and CADM1, followed by appropriate secondary antibodies. All antibodies used were listed in Supplementary Table 3. The slides were mounted with ProLong Gold Antifade Reagent containing DAPI, and scanned using Vectra® Polaris™ Imaging System (Akoya Biosciences). Images were analyzed by Image J software (National Institutes of Health, USA).

### Immunofluorescence staining

Immunofluorescence (IF) staining was performed as previously described [14]. Cells on coverslips were fixed, permeabilized and followed with an overnight incubation of primary antibodies (Supplementary Table 3) at 4 °C. On the following day, cells were incubated with Alexa Flour 488 or Alexa Flour 594-conjugated donkey anti-mouse/rabbit secondary antibodies (Thermo) at room temperature (RT) for 30 min. Cell nuclei were counterstained with Hoechst 33342 (Thermo). The staining was visualized using an EVOS FL microscope (Life Technology, Gaithersburg, MD, USA) or a laser scanning confocal microscope (Leica Microsystems GmbH, Mannheim, Germany).

### Flow cytometric assay

CD133 positive cells were measured by flow cytometer. Briefly, cells (1 × 10^6^) were washed and incubated with fluorescence labeling antibodies (PE mouse anti-CD133, clone AC133, Miltenyi Biotec; FITC mouse anti-CD44, clone C26, BD) at RT for 30 min. Samples were washed, assayed via FACS Canto II (BD) immediately, and analyzed secondarily using Diva software (BD).

### Western blot analysis

Protein was extracted using RIPA lysis buffer (Cell Signaling Technology, Danvers, MA, USA). The concentration was quantified by the BCA Protein Assay Kit (CWBIO, China). Equal amounts of proteins were loaded onto 10% polyacrylamide gel and separated by SDS-PAGE. Samples were then transferred to PVDF membranes. After blocking with 5% non-fat milk, the membranes were incubated with primary antibodies and secondary antibodies. Target bands were visualized using chemiluminescence (Millipore, Billerica, MA, USA). Antibodies used to determine the indicated proteins are specified in Supplementary Table 2.

### Cellular uptake assay

Cellular uptake profiles were measured by flow cytometry. Briefly, cells were seeded into six-well plates for 24 h. After incubation with or without Dox (10 μM) for 3 h, cells were harvested and washed with cold PBS. Cells without Dox incubation were used as background controls. Dox uptake was detected by flow cytometer and analyzed with FlowJo VX software (RRID:SCR_008520, FlowJo, LCC, OR, USA). The mean fluorescence intensity (MFI) ratio of Dox (+) to their blank control was used as relative MFI.

### Tumor sphere formation and growth assay

Tumor sphere formation was analyzed as described previously [20]. Briefly, cells were seeded in 6-well plate (5000 cells/well) and maintained in NSC culture medium (DMEM/F-12 + 20 ng/ml of bFGF (PeproTech, Rocky Hill, NJ) + 20 ng/ml of EGF (PeproTech) + N2 (Thermo) + B27 (Thermo) and allowed to form spheres. Photographs of tumor spheres were taken at the indicated time points and sphere diameter was measured. At least 10 random fields were analyzed per experiment.

### Limiting dilution analysis (LDA)

Glioma spheres were dissociated into single-cell suspensions using Accutase cell dissociation Reagent (Millipore, Billerica, MA) and plated in 96-well low attachment plates (Corning, NY) by a limiting dilution fashion at 5, 20, 50, 100, 200 cells per well and held in NSC culture media. Fresh medium was added every 3–4 days by removing 50% of the old medium and replacing it with fresh medium. After 2-3 weeks, each well was examined for the formation of tumor spheres. Clonal frequency and significance were analyzed using the Extreme Limiting Dilution Analysis (http://bioinf.wehi.edu.au/software/elda/).

### Statistical analysis

All experiments were independently repeated at least three times, and the data were expressed as mean ± standard deviation (SD). All data were analyzed with the SPSS 20.0 software package (IBM Corp.) and consistent with the normal distribution and the homogeneity variance test. A *t*-test was used to compare the significant difference between two groups. One-way ANOVA was used for the mean comparison between multiple sample groups, and the Tukey ad hoc test was used for intra-group multiple comparisons. *P* < 0.05 was considered statistically significant. Graphpad Prism 6.0 software was used for image analysis.

## Results

### Construction and confirmation of an acquired TMZ-resistant GBM xenograft model

To obtain in vivo acquired TMZ-resistant GBM cells, we implanted U87 cells into nude mice and performed three cycles of TMZ treatment along with tumor passage in vivo using a mild concentration of TMZ (Fig. 1A). Temozolomide-resistant tumor tissues (TMZ-R) or cells were isolated from xenografts of serially treated mice. The parental tissues and cells isolated from saline-treated xenografts were named TMZ-S. These cells and tissues were frozen for subsequent in vitro and in vivo studies. Secondary xenograft models generated from the isolated frozen cells showed that tumors derived from TMZ-R cells grew more rapidly than those derived from TMZ-S cells (Fig. 1B). While in the presence of TMZ, TMZ-R tumors exhibited a loss response to chemotherapy, relative to the rate of DNA amplification of TMZ-S tumors (Fig. 1C). Moreover, chemotherapy-treated TMZ-R tumors showed comparable growth ability with saline-treated TMZ-S tumors (Fig. 1D). Compared with parental cells, TMZ-R generated cells showed impaired response to various anticancer drugs, as confirmed by an increased IC50 (Fig. 1E), indicating the acquisition of multidrug resistance. Two typical multidrug resistance-associated protein-coding genes, *ABCB1* (*MDR1*) and *ABCC3* [21, 22] were markedly upregulated in TMZ-R tumors and derived primary cells (Fig. 1F), revealing that drug efflux pumps may play an important role in mediating TMZ-resistance. DOX, a red fluorescent molecule, was used to determine whether drug efflux pump downregulation resulted in reduced drug accumulation in TMZ-R cells. The flow cytometry assay showed that the uptake of DOX by TMZ-R cells decreased significantly (Fig. 1G), confirming chemotherapy resistance is associated with impaired drug uptake [22, 23]. Consistent with the previous finding in U87 resistant cells [10, 24], continuous TMZ treatment failed to reduce the hypermethylation of MGMT promoter or induce re-expression in U87 cells (Fig. 1H, I), demonstrating that MGMT expression/methylation were not contributors to TMZ-resistance of U87 in vivo. Therefore, the established U87 TMZ-resistant xenograft provides a stable acquired TMZ-resistance in vivo model, subsequently yielding stable, multi-drug resistant derived primary cells.

### The acquired TMZ-resistant tumor contains more quiescent cancer stem cells

**Fig. 2:**
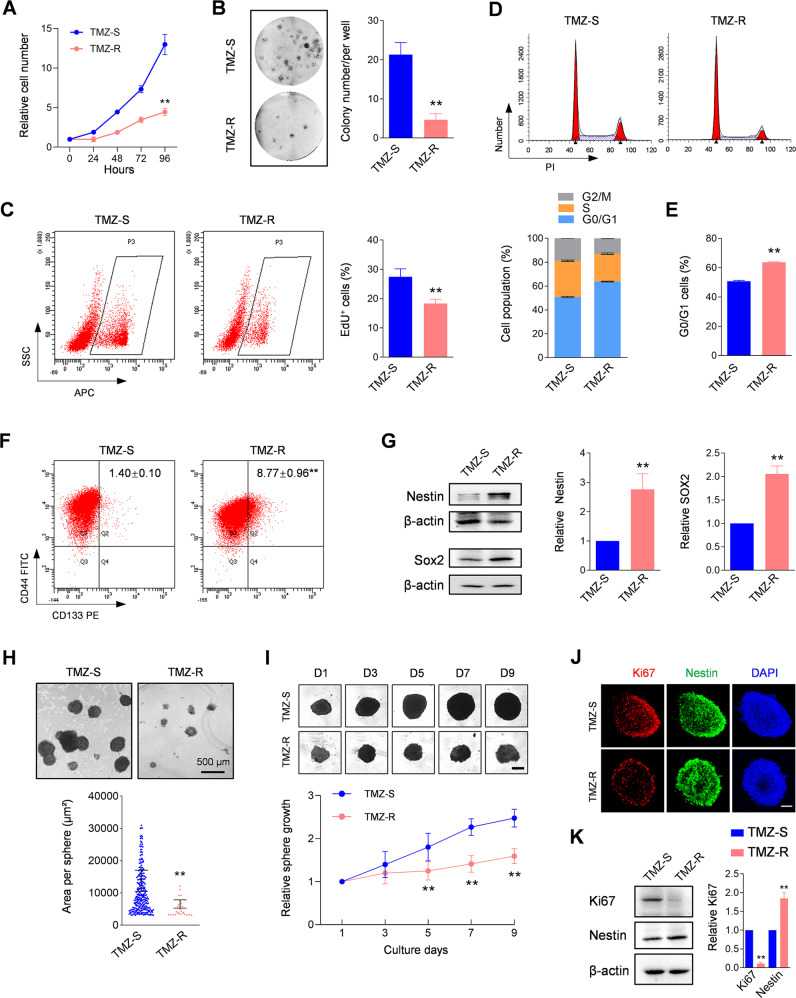
The acquired TMZ-resistant tumor has more quiescent cancer stem cells. **A** Cell growth assay of indicated cells (*n* = 6, **p < 0.01). **B** Colony assay of indicated cells (*n* = 3, **p < 0.01). **C** EdU incorporation assay detecting the DNA replication in indicated cell. The EdU labeling cells were analyzed by flow cytometry (left) and the statistical result was indicated in the right panel (*n* = 3, **p < 0.01). **D**. Cell cycle was detected by flow cytometry (upper) and the statistical results were shown in the lower panel. **E** Relative population of cells in G0/G1 stage were quantified (*n* = 3, **p < 0.01). **F** Flow cytometry assay indicating the percentage of CD133 positive cells in the indicated cells (*n* = 3, **p < 0.01). CD44 severed as a common marker for U87. **G** Western blot analysis showing the expression of Nestin and SOX2 in the indicated cells (*n* = 3, **p < 0.01). β-actin served as a loading control. **H** Sphere formation assay of the indicated cells. The number and area of the spheres were analyzed (**p < 0.01). Bars, 500 μm. **I** 3D-tumor spheroid growth was continuously recorded at the indicated time (upper) and quantitatively analyzed (lower; *n* = 8, **p < 0.01). Bars, 200 μm. **J**. Immunofluorescence (IF) staining of spheroids using the indicated antibodies. DAPI labeling of the nuclei was performed. Bars, 100 μm. **K** Western blot assay showing the expression of Ki67 and Nestin in spheroids derived from the indicated cells (**p < 0.01). β-actin served as a loading control.

Cell cycle arrest or muted cell cycle progression allows extensive DNA repair mechanisms which contribute to chemoresistance in cancers [25–27]. Consistent with the previous finding that in vitro U87 TMZ-resistant cells display cell cycle arrest [10], the assays in this study showed that primary cells derived from in vivo TMZ-R tumors exhibited lower proliferation compared to the primary cells derived from TMZ-S tumors (Fig. 2A, B). EdU incorporation further indicated that DNA replication in TMZ-R tumor-derived cells was decreased significantly (Fig. 2C). Additionally, cell growth inhibition of TMZ-R cells was associated with cell cycle arrest (Fig. 2D), and the ratio of G0/G1 cells in TMZ-resistant cells increased significantly (Fig. 2E). The presence and increase of quiescent cancer stem cells (CSCs) are considered as a major contributor of tumor recurrence after treatment resistance [28, 29]. These quiescent CSCs exhibit lower self-renewal and proliferation capacity, leading to the escape from conventional chemotherapy [30]. The current study also demonstrated that the number of CD133 positive (CD133^+^) cells was markedly increased in TMZ-R tumors (Fig. 2F), indicating TMZ-continuous chemotherapy drives an expansion of stem cell properties. This observation was further verified in the TMZ-R tumors by molecular profiling which identified higher expression of Nestin and SOX2, two classical neural stem cell markers (Fig. 2G). Interestingly, CSCs spheres derived from TMZ-R in 3D spheroid culture were much smaller than those derived from TMZ-S cells (Fig. 2H). The limiting dilution assay (LDA) further confirms the lower CSCs sphere formation efficiency of TMZ-R cells (Supplementary Fig. 1A). Upon closer inspection, the derived TMZ-R CSCs spheres exhibited markedly lower amplification ability (Fig. 2I), and sparse distribution of Ki67 (Fig. 2J), which widely serves as a proliferation marker for human tumor cells [31]. The significant decrease of Ki67 expression in TMZ-R CSCs was further confirmed in the overall analysis (Fig. 2K). Collectively, these results support that TMZ-R-derived CSCs exhibit quiescent properties.

### scRNA-seq identifies a subgroup of cancer stem cell in TMZ-resistant tumors

**Fig. 3:**
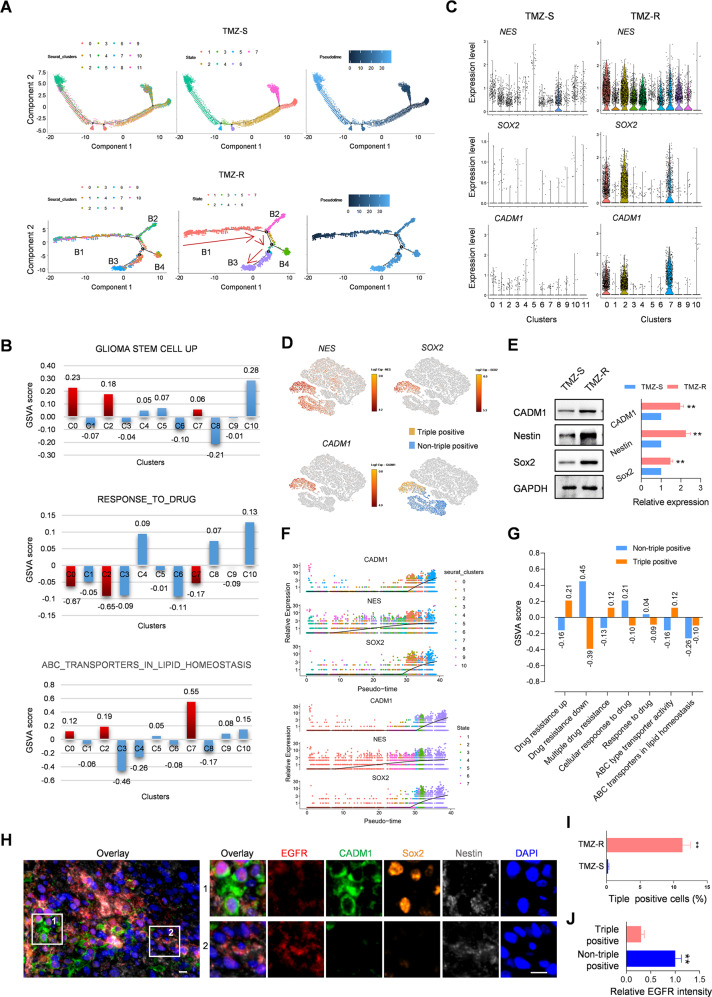
scRNA-seq analysis identified a subgroup of cancer stem cells in TMZ-resistant tumors. **A** Pseudotime analysis based on the single-cell RNA-seq. The indicated cell clusters were placed on the trajectory according to sequential transcriptional changes (left), and re-grouped into different states (middle) and time course (right). **B** GSVA analysis displaying cellular behavior difference among cell clusters in TMZ-R. Bar charts visualizing GSVA scores of indicated gene sets in GO-BP terms. **C** Enrichment analysis of *NES*, *SOX2*, and *CADM1* in cell clusters derived from TMZ-S and TMZ-R. **D** LOUPE browser analysis displaying the distribution of *NES*, *SOX2*, and *CADM1* in 20 subgroups identified by Graph-based clustering. **E** Western blot assay showing the expression of indicated proteins in the indicated tumors (**p < 0.01). GAPDH served as a loading control. **F** Pseudotime analysis showing the cellular distribution according to the expression of *SOX2*, *CADM1* and *NES* along the pseudotime axis. **G** GSVA analysis displays the enrichment of upregulated genes in the indicated gene sets. **H** Representative mIHC/IF images showing the expression of triple markers (Nestin/Sox2/CADM1) and EGFR in TMZ-R tissue. Bars, 10 μm. **I** The proportion of triple positive cells (Nestin^+^/Sox2^+^/CADM1^+^) in TMZ-S and TMZ-R tissue (*n* = 6, **p < 0.01). **J** The quantitative analysis of EGFR fluorescent intensity in triple positive cells and non-triple positive cells in TMZ-R tissues (*n* = 6, **p < 0.01).

Next, a microfluidic-based approach of the 10× Genomics® platform was executed to obtain the transcriptome of cells isolated from TMZ-S and TMZ-R tissues according to the established method [32]. A total of 18,894 cells and 35,884 reads per cell were estimated in TMZ-S cell library, and the mean genes per cell were 2776. In TMZ-R cells, 8627 cells and 73,198 reads were obtained, which resulted in a mean gene per cells at 3669 (Supplementary Fig. 2), suggesting a robust alteration of gene transcription in TMZ-R tumors. Using the Graph-Based clustering analysis, we identified a genomic heterogeneity between TMZ-S and TMZ-R-derived cells (Supplementary Fig. 3A). Overall, 11 distinct clusters in TMZ-R cells and 12 in TMZ-S cells were identified via t-distributed stochastic neighbor embedding (t-SNE). The SingleR annotation indicated an alteration of cytological diversity between TMZ-S and TMZ-R, and disclosed a marked increase of tissue stem cell populations in TMZ-R derived cells as compared with TMZ-S derived cells (20.93% vs 3.90%; Supplementary Fig. 3B). To further explore the heterogeneity and evolution of drug resistant cells, a pseudotime analysis was performed based on the single-cell RNA-seq data (Fig. 3A). The derived cell clusters were placed on the trajectory according to sequential transcriptional changes (left), and re-grouped into different states (middle) and time course (right) by Monocle. Compared to TMZ-S, the reconstructed trajectory of TMZ-R derived cells exhibited more branches (denoted “B1”, “B2”, “B3”, and “B4”). Notably, in TMZ-R cells, the root of trajectory (B1) was populated by the majority of cluster 4, 5, 9, and 10, and the branches (B3, B4) was populated by the majority of cluster 0, 2 and 7. Next, we identified the top 50 genes in TMZ-R with branch-dependent expression (qval <1e-4) for branch points 2 and 1, respectively (Supplementary Fig. 4), which may play an important role in determining the diverse evolution of cell fate induced by TMZ. Next, gene set variation analysis (GSVA) was applied to explore the cellular behavior of cell clusters in TMZ-R with differential transcriptional profiles. According to the GO-BP gene sets, 6 of 11 clusters in TMZ-R enriched in glioma stem cells were upregulated, among which clusters 0, 2, 7 exhibited a lowered drug response and ABC transporter upregulation (Fig. 3B). Although TMZ-S also showed enrichment of glioma stem cell clusters, only C8 exhibited diminished drug reactivity, and only C5 displayed upregulated drug transport (Supplementary Fig. 5). These results provide insight into the functional diversity of CSCs induced by recurrent TMZ treatment, especially their diminished drug reactivity. Therefore, the subsequent cellular assays were focused on clusters 0, 2, and 7 in TMZ-R cells. To label these cells, human brain cancer stem cell markers were identified from the Human Cell Atlas Database and the existing literature (Supplementary Table 4). These reference genes were subsequently applied for screening. qRT-PCR analysis was used to screen 8 markers, noting their increase in TMZ-R tumors, including *PROM1* (*CD133*), *SOX2*, *NES* (*Nestin*), and *CD44* (Supplementary Fig. 6). Moreover, we found that three stem cell markers, *NES (Nestin)*, *SOX2* and *CADM1* were particularly enriched in CSC clusters 0, 2, and 7 (Fig. 3C). Among these three markers, *SOX2* and *CADM1* were significantly enriched in clusters 0, 2, and 7. Further analysis using Graph-based clustering identified 20 additional subgroups based on cluster-specific genes and biological processes derived from a total 27,521 cells spanning TMZ-R and TMZ-S libraries (Supplementary Fig. 7). Results indicated that cells from TMZ-R and TMZ-S tumors were differentially distributed. LOUPE browser analysis confirmed that *NES* was widely expressed in all clusters overall, but especially overexpressed in clusters of TMZ-R-derived CSCs. Moreover, we found that *SOX2* and *CADM1* were enriched in a particular subgroup of TMZ-R cells, distinguishing a triple positive (*SOX2*^+^/*CADM1*^+^/*NES*^+^) CSC subgroup derived from TMZ-R tumors (Fig. 3D). This molecular profile was additionally observed upregulated at the protein level in TMZ-R tumors (Fig. 3E). The existence of triple positive cells in TMZ-R tumors and TMZ-S was identified by mIHC/IF staining, and almost no triple positive cells were observed in TMZ-S cells (Fig. 3H, I). Highly expressed in U87 cells [33] and widely expressed in clusters of TMZ-R cells, *NES* is used as a broad-spectrum marker of U87 cancer stem cells. Combined with *SOX2* and *CADM1, SOX2*^+^/*CADM1*^+^/*NES*^+^ served as a genomic signature for a subtype of CSCs derived from TMZ-R tumors. The triple-positive gene signature was traced using pseudotime analysis for evaluation of cellular evolution. As shown in Fig. 3F, the expression of *SOX2*, *CADM1* and *NES* in individual cells was distributed along the pseudotime axis. These cells were enriched in clusters (upper) and stats (lower) according to the cellular evolution in pseudotime. *SOX2*, *CADM1*, and *NES* triple-positive cells accumulated at the terminal, suggesting that recurrent TMZ treatment induces a de-differentiation process to induce the triple positive CSCs. Based on this evidence, the cells derived from TMZ-R were divided into two sub-groups according to the median expression of each of the individual genes (*SOX2*, *CADM1* and *NES*) to distinguish between triple positive cells (*SOX2*^+^/*CADM1*^+^/*NES*^+^) and non-triple positive cells. Next, the CSCs with the upregulated genes were collected and GSVA was applied to comparatively evaluate the drug response and cellular phenotypes of the two sub-groups. We demonstrate that triple-positive cells exhibited more advantages in drug resistance, which is possibly associated with the enhancement of ABC transporter activity (Fig. 3G). Overall, our findings suggest that recurrent TMZ treatment induces a diverse cellular evolution which induces a unique subgroup of CSCs defined by a lowered drug response, a major contributor to TMZ resistance.

### The identified cancer stem cell subgroup displays low expression of EGFR

**Fig. 4:**
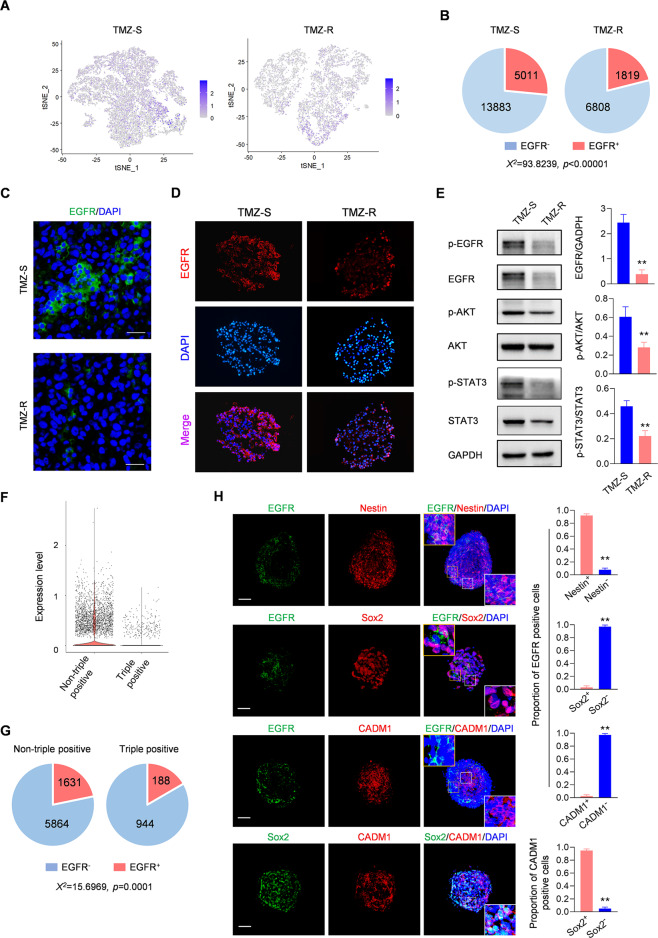
The identified cancer stem cell subgroup contains low expression of EGFR. **A** The LOUPE browser analysis shows the EGFR expression in the indicated cells. **B** The pie plots show the number of cells according to EGFR expression between TMZ-S and TMZ-R (Fisher’s exact test). **C** IF staining showing the EGFR expression in indicated xenografts. DAPI labeling of the nuclei was performed. Bars, 100 μm. **D** IF staining showing the EGFR expression in GSC spheres derived from indicated cells. DAPI labeling of the nuclei was performed. Bars, 100 μm. **E** Western blot assay comparing the expression of the indicated protein in TMZ-S and TMZ-R tumors (*n* = 3, **p < 0.01). GAPDH served as a loading control. **F** The bioinformatics analysis showing the expression pattern of EGFR between non-triple positive cells and triple positive cells in TMZ-R. **G** The pie plots show the number of cells according to EGFR expression between non-triple positive cells and triple positive cells in TMZ-R (Fisher’s exact test). **H** Representative images of double immunofluorescence (IF) staining of EGFR and Nestin, Sox2, or CADM1 in the cultured 3D spheroids derived from TMZ-R cells. DAPI labeled the nuclei. The right panel showing the proportion of co-expression cells (*n* = 8, **p < 0.01). Scale bars, 50 μm.

Low yield of stem cell spheres and the quiescent state of TMZ-R-derived CSCs hinted at a low response to nutritional factors in stem cell culture medium. Given that EGFR is responsible for the maintenance and self-renewal of CSCs [34], we speculated that TMZ-R-derived CSCs may express lowered EGFR. LOUPE browser analysis indicated that EGFR was scattered in TMZ-R tumor cells (Fig. 4A), determining that the number of cells above the cutoff of EGFR expression in TMZ-R was significantly less than in TMZ-S (Fig. 4B). Accordingly, immunofluorescence staining confirmed the scattered expression of EGFR in TMZ-R tumors (Fig. 4C) and CSC spheres (Fig. 4D). Western blot assay further confirmed an overall EGFR downregulation in TMZ-R tumors, and the decrease of downstream AKT and STAT3 phosphorylation (Fig. 4E). Next, we examined the expression of EGFR in triple positive cells derived from TMZ-R tumors. The bioinformatics analysis indicated the expression pattern of EGFR between non-triple positive cells and triple positive cell in TMZ-R (Fig. 4F). It disclosed that triple-positive cells have lower EGFR level as compared to non-triple positive cells (Fig. 4G), which was further directly observed in TMZ-R tumors that EGFR expression was significantly lower in triple positive cells than it in non-triple positive cells (Fig. 3H, J). In the cultured 3D spheroids derived from TMZ-R, the double IF staining showed that Nestin occurred in most EGFR-positive cells, indicating Nestin worked as a common marker for these derived spheroids. Moreover, it revealed that Sox2 and CADM1 were co-expressed in high proportion. However, EGFR and Sox2 or CADM1 was mutually exclusive (Fig. 4H). Collectively, these data suggest that EGFR deficiency underlies the quiescent state of TMZ-R cells, especially in the triple-positive subgroup.

### EGFR deficiency confers CSCs cell cycle arrest and acts as an anti-chemoresistance target

**Fig. 5:**
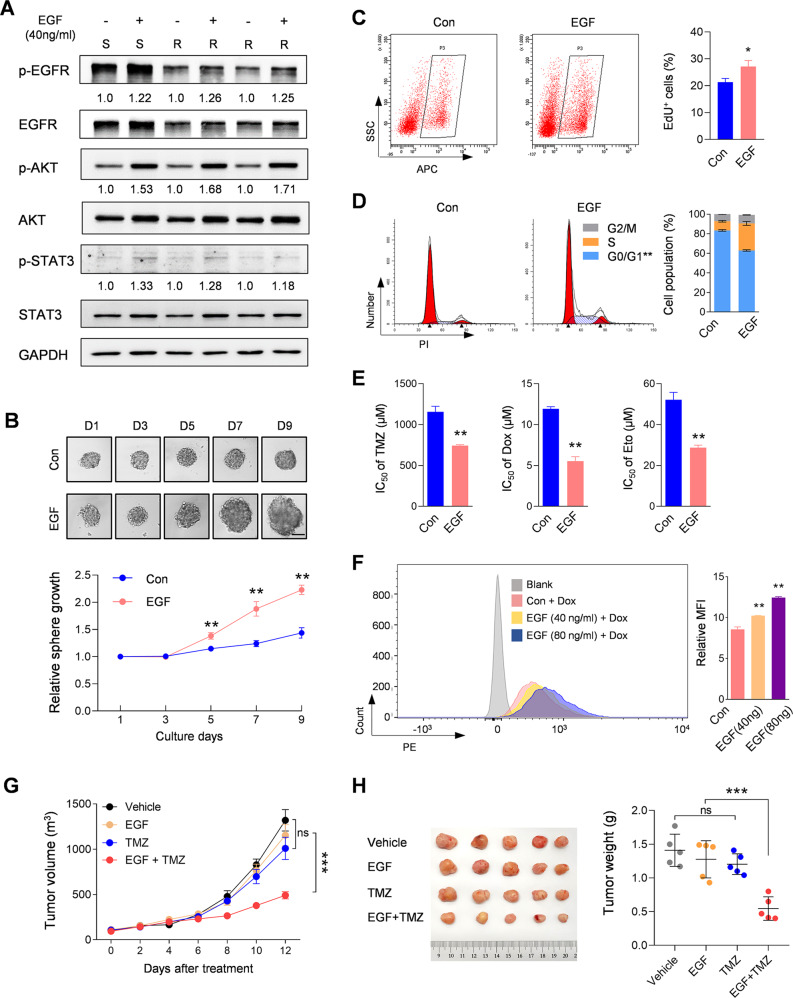
EGFR deficiency confers CSCs cell cycle arrest and chemoresistance. **A** Western blot analysis showing the activation of EGFR, AKT, and STAT3 in TMZ-S and TMZ-R cells upon EGF stimulation. The relative quantification of EGFR, AKT, and STAT3 phosphorylation was listed under the bands. GAPDH served as a loading control. **B** 3D-CSC spheroid growth was continuously recorded at the indicated time (upper) and quantitatively analyzed (lower; *n* = 8, **p < 0.01). Bars, 200 μm. **C** The EdU labeling cells were analyzed by flow cytometry (left) and the statistical result were indicated in the right panel (*n* = 3, **p < 0.01). **D** Cell cycle was detected by flow cytometry (left) and the statistical results were shown in the right panel (*n* = 3, **p < 0.01). **E** IC50 assay indicating drug response alteration of TMZ-R cells upon EGF stimulation (*n* = 3, **p < 0.01). **F** Flow cytometry (FC) assay (left) and the statistical analysis of MFI (right) showed the alteration of DOX uptake by the indicated cells upon EGF stimulation (*n* = 3, **p < 0.01). **G** Tumor growth assay of the indicated xenografts after the first TMZ injection. Mice were subcutaneously injected with TMZ-R cells and subsequently received intragastric administration of TMZ (20 mg/kg) combined with an intratumoral multipoint injection of EGF (25 μg/kg) every two days after the tumors reached 100 mm^3^. Tumor volume was monitored as indicated day from the 1st TMZ injection. Xenografts were collected at the 12nd day post TMZ treatment. **H** Representative images of subcutaneous xenografts collected at the 12th day after treatment (left), and the tumor weight analysis (mean ± SD, *n* = 5, **p < 0.01).

Although containing lower EGFR expression, CSCs derived from TMZ-R tumors possessed the ability to respond to EGF stimulation, which was reflected in the phosphorylation of EGFR and downstream activation of Akt/STAT3 induced by EGF stimulation (Fig. 5A). Accordingly, the amplification ability of CSCs sphere derived from TMZ-R tumors was restored under the continuous stimulation of EGF (Fig. 5B), as further evidenced by the recovery of self-renewal capacity indicated in an increased of EdU positive rate and normalized 3D sphere diameter on the final culture day (Fig. 5C). The limiting dilution assay confirms that CSCs sphere formation efficiency of TMZ-R cells was restored after continuous EGF incubation (Supplementary Fig. 1B). Moreover, continuous EGF treatment led to a significant decrease in the proportion of cells in G0/G1 phase (Fig. 5D), revealing that EGF treatment induces a release of quiescent cells from cell cycle arrest. These results suggest that EGFR is vital for cellular quiescence regulation, leading us to explore whether the EGF-stimulated cell cycle rescue was conducive to improving the chemosensitivity of TMZ-R. The IC50 assay (Fig. 5E) provided evidence that EGF treatment improved the in vitro chemosensitivity of TMZ-R cells. Flow cytometry assay further indicated the effect of EGF treatment resulted in an enhancement of drug uptake in TMZ-R cells (Fig. 5F). Based on these in vitro findings, we next determined whether EGF is a viable means to overcome chemoresistance in vivo. The xenograft model using frozen TMZ-R cells was treated with a combination therapy of TMZ and EGF for 9-days post implantation. The mice were injected i.p. once every 2 days with TMZ (20 mg/kg) combined with an intratumoral multipoint injection of EGF (25 μg/kg) in the span of 12 days. As shown in the tumor growth curve (Fig. 5G) and the weight assay of collected tumors on the final day (Fig. 5H), EGF alone did not alter the tumor growth ability, but combination therapy resulted in a significant inhibition of tumor growth in vivo. These results suggested that EGF improves the sensitivity of TMZ-R tumors to TMZ, implicitly pointing to the deficiency of EGFR signaling in the endowment of CSC chemoresistance in glioblastomas in an MGMT-independent context.

## Discussion

The acquisition of TMZ resistance is a major clinical challenge for GBM treatment, and related therapeutic strategies to overcome such acquired TMZ resistance are far from optimal. This makes it particularly important to understand the mechanisms leading to acquired resistance. Cancer stem cells account for much of the chemo and radiotherapy resistance associated with GBM. The characteristics of these cells have been extensively studied. Among several gene targets, the DNA repair enzyme O^6^-methylguanine DNA methyltransferase (MGMT) has been widely implicated in conferring drug resistance of CSCs [35], so that many clinical studies have lauded MGMT expression/promoter methylation as a prognostic indicator of TMZ-treatment [36]. However, many MGMT-deficient patients remain impacted by TMZ resistance, indicating a more complex mechanism of acquired resistance in these cells. The focus of this study was to clarify the cellular evolution of MGMT-null, primary CSCs obtained from a stable, acquired TMZ-resistant GBM xenograft model. Additionally, we sought to identify a therapeutic target for improving the chemosensitivity of TMZ-R cells.

The current study provides direct evidence that cancer stem cells are closely linked to chemoresistance and reveals that TMZ chemotherapy induces cellular evolution resulting in complex heterogeneity and enrichment of glioma stem cells. Using scRNA-seq and pseudotime analysis, we and other investigators have demonstrated the evolution of cancer stem cells as an important product of this adaptive response to TMZ [37]. While the aberrant events that trigger CSC transformation in TMZ-treated glioblastomas are not entirely worked out, studies in which patient-derived GBM cell lines have been successfully reprogrammed into CSCs through forced expression of neural stem cell transcription factors (Oct4, Sox2, Nanog) lend credence to the notion of aberrant de-differentiation [38]. An abundance of evidence points to the role of extra-cellular factors, cell adhesion molecules, Notch, Wnt/β-catenein, BMP, and TGFβ signaling in the development of chemotherapy-resistant, quiescent CSCs to name a few [38–41]. Despite this, it is increasingly clear that the transformation of CSC is complicated by the existence of divergent CSC subtypes with unique genetic signatures and non-uniform profiles of chemo and radiotherapy resistance [42]. On the other hand, some investigators have provided evidence for the notion that quiescent cancer stem cell populations are not created, but lay dormant and become “reawakened” in response to cytotoxic chemotherapy [43].

The finding of a low proliferation phenotype and the activation of drug-efflux-like ATP binding cassette family transporters represent typical features of CSCs which support their survival under chemotherapy pressure [44]. However, the inconsistencies among the observed proliferative potential of TMZ-R CSCs in vivo and in vitro remain a problem, and may be rooted in the differences of an in vitro versus in vivo environment [45, 46]. While the single in vitro environment is convenient for TMZ-R cells to maintain a lasting quiescent state, the complexity of the in vivo microenvironment almost certainly implicates additional pressures which shape the adaptive cell response, and therefore, the maintenance of the quiescent state of chemo resistant cells. Toward the aim or reconciling the two models, one research team developed a 3D GBM organoid from primary GBM patient cells, leveraging gene editing tools to insert an inducible proliferation reporter that more closely recapitulates drug resistance in a heterogenous tumor model. Similar to our findings, quiescent GBM cells lacking proliferation markers were tracked to just 0.2–1% of organoid cells after just 4 weeks of recurrent drug treatment [39]. Additionally, these investigators observed a reduced sphere diameter in the quiescent GBM cells compared to spheres formed by non-quiescent primary cells.

Undifferentiated (or de-differentiated) glioma stem cells are a unique subset of therapy-resistant cells that express markers of stemness, including CD133^+^ and neural stem cell markers like nanog, Sox2, and Nestin [47]. The drug resistance of these cancer stem cells underlies a majority of the recurrence of tumors and is thus an essential property to characterize. In this study, a subset of CSCs expressing the triple positive signature *NES*^+^/*Sox2*^+^/*CADM1*^+^ demonstrated the greatest features of drug resistance. The expression of the neural stem cell markers Nestin and Sox2 are consistent with the features of most drug-resistant glioma stem cell populations and support the neural stem cell lineage of GBM. However, the role of CADM1 in quiescent CSCs displaying TMZ resistance has been less studied. One study found that high serum levels of exosomal miR-148a (a target of CADM1) existed in GBM patients compared to healthy controls [48]. They further found that inhibiting the miRNA not only increased the expression of CADM1 but inhibited activation of the STAT3 pathway, a determinant of GBM proliferation and tumorgenicity. A similar study in ovarian cancer cells described the downregulation of the CADM1-targeting miR-486, which saw reduced cell viability, proliferation, and an increased proportion of cells in G0/G1 arrest [49]. Moreover, CADM1 was inversely proportional to the expression of cyclin D1, cyclin E, CDK6, and p21. The authors of that study further demonstrated that reductions in cell viability, invasion, proliferation, increased apoptosis rate, and G0/G1 arrest were specific to the miR’s action on CADM1 as the combined application of an inhibitor of miR-486 and an si-CADM1 normalized all anti-tumorigenic features of CADM1-expressing cells. The findings of the referenced studies are similar to the characteristics observed in the triple-positive quiescent stem cells derived from TMZ-R tumors in the present study and align with a CADM1 + phenotype. Together, our studies corroborate a role for CADM1 as a tumor suppressor, regulating proliferation and keeping tumorgenicity at bay. Evidence from other cancer types has further implicated CADM1 in the repression of tumorgenicity through the negative regulation of G1/S transition [50], which is consistent with the proportion of triple positive CSCs observed in G0/G1 arrest herein.

The present study found a lowered expression of EGFR in drug-resistant, quiescent CSCs derived from TMZ-R tumors. Our findings corroborate EGFR expression and activity patterns observed in several TMZ-resistant cell lines and GBM patient tumor tissues [51]. For example, Areeb et al report that in addition to deficient EGFR expression in treatment-resistant cells, there is significant upregulation of the EGFR-targeting miR-221, which likely serves as a mechanism of reduced EGFR expression. The present findings also compliment a previous study wherein pharmacological inhibition of EGFR induced a population of quiescent GSCs expressing similar neural stem cell markers [52]. In contrast, one study found that the human EGFR inhibitor LRIG1 can regulate quiescent neural stem cells’ responsiveness to EGFR signaling (prompting NSC exit from quiescence) [53], while another reported that LRIG1 can reverse the course of multi-drug resistance by suppressing ABCB1 and ABCG2 [54] in glioblastoma cell lines. The latter studies support a role for EGFR/Akt/STAT3 signaling in conferring tumorgenicity [55] and sensitivity to temozolomide, which is at odds with our current findings. The discordance is likely rooted in the MGMT context of the different experimental models. For example, while TMZ resistance was associated with upregulation of STAT3 in the GBM cell line U87 [56, 57], this was dependent on concurrent upregulation of the DNA repair enzyme MGMT. In contrast, activation of AKT/STAT3 and upregulation of the EGFRvIII variant (present in 1/3 of GBMs) were associated with TMZ sensitivity but only in patients with highly methylated MGMT promoters (associated with loss of MGMT expression) [58]. Presented in the context of an MGMT-null GBM model, our findings of reduced EGFR expression and downstream AKT and STAT3 activation in quiescent and TMZ-resistant GSCs complement the findings of Struve et al and point to an important consideration across all GBM research.

Finally, we report that the decreased proliferation, cell cycle arrest, and sphere growth characteristic of the TMZ-R-derived CSC subpopulation were all reversible by EGF treatment. Moreover, the reduced drug response of these cells was reversed by EGF. In vivo, EGF-treated TMZ-R animals displayed a more normalized tumor volume/weight only when EGF was paired with TMZ treatment, but not by EGF alone, suggesting that while EGF can help quiescent CSCs revert to their differentiated state and the associated tumorigenic phenotypes, it cannot act alone as an effective glioblastoma therapeutic. The finding that deficient EGFR expression in TMZ-R-derived CSCs can be overridden by exogenous EGF supports the notion that the cellular phenotypes may be driven by reduced activation of EGFR signaling due to diminished expression of available receptors. In such a case, overwhelming the cells with EGFR ligand can increase the likelihood of binding/activation. Alternatively, one study has reported that exogenous EGF can stimulate the expression of NTN4, a regulator of GBM tumor progression/proliferation via ITGB4-Akt signal activation [59]. This is consistent with our observations that EGF treatment restored phosphorylated Akt in tandem with restored tumorigenic phenotypes. The Li et al study also lends support to the concept of redundancy along the EGFR/Akt/STAT3 pathways, which may incidentally explain the clinical ineffectiveness of various EGFR-targeting therapies to date [55]. Combinatorial, EGFR-targeted treatment has previously been shown to increase the efficacy of TMZ in GBM cell lines and animal-derived tumors [60]. While the aberrant EGFR expression in that study was not a downregulation of wild-type EGFR, rather expression of a common glioblastoma mutation (EGFRvIII), it supports the idea that specific cohorts of GBM patients may benefit from combined EGFR-targeting and TMZ therapy. It is worth noting that EGFR-targeted therapy is not the only treatment that aids in the re-sensitization of TMZ-R cells. Another study treated TMZ-R GBMs with the cytotoxic agent with aferin A to inhibit MGMT expression [61]. Combined withaferin A and TMZ treatment overcame the GBM’s chemoresistance and potentiated the efficacy of TMZ, similar to our findings. Collectively, our study and the existing literature support the idea that TMZ resistance of GBM tumors is multi-dimensional, likely rooted in a variety of signaling cascades and molecular events resulting in diverse cellular phenotypes which shape the drug response.

One of the limitations of this study was the subcutaneous tumor model, which may not accurately recapitulate the CNS microenvironment that shapes the course of glioblastomas clinically [62]. On the other hand, the drug-resistant cells obtained after acclimation continue to demonstrate drug-resistant properties in situ. Taken together, our model lends itself to repeated generation of stable, drug-resistant cells. Indeed, serial propagation in vivo is the defining characteristic of CSCs, as opposed to any particular molecular signature observed in vitro [63]. Toward that aim, another advantage of this model was the in vivo serial transplantation which resulted in a validated phenotype/genotype of CSCs. The nature of the model also circumvented many of the challenges observed in preclinical models which do not allow for the repopulation of tumors by surviving cancer stem cells nor the enrichment of repopulating clones due to long-term continuous chemotherapy or single-dose treatment [43]. In future studies, it will be important to thoughtfully investigate whether recurrent chemo and radio therapy truly confer the genotypes and phenotypes observed in drug-resistant quiescent CSCs or whether these subsets constitutively exist and drive the responsiveness of the tumor to treatment. Additionally, it will be pertinent to evaluate the patterns of these CSCs subsets across diverse glioblastoma patient types and characterize their plasticity throughout the course of therapeutic manipulation [63]. Finally, future efforts should continue to explore cross-talk between quiescent CSCs and adjacent non-CSCs, which have demonstrated negative feedback loops which favor the survival of oncogenic NSCs to promote tumorigenesis [40].

Despite the availability of GBM treatments, patient survival remains between 12-15 months due to tumor recurrence largely driven by treatment-resistant cells [2]. This study aimed to characterize a unique subset of quiescent, TMZ-R-derived CSCs using bioinformatics, genomics, and cellular assays. We describe the stable derivation of primary tumor cells which display the classical features of multi-drug resistant, quiescent CSCs, including reduced proliferation capacity, cell cycle arrest, diminished drug uptake, and lower amplification ability in 3D culture. Further, we identified a niche of CSCs which was particularly advantageous for evading responsivity to TMZ. The molecular signature of this subset included elevated expression of the canonical neural stem cell markers Nestin and Sox2, along with the elevated expression of CADM1. This triple-positive CSC subgroup additionally observed diminished expression of EGFR and downstream signaling which was overcome by exogenous EGF. We conclude that diminished EFGR activity in the context of MGMT-null organisms, may underlie the acquisition of TMZ resistance and quiescence of CSCs in recurrently-treated glioblastoma. In turn, restoration of EGFR signaling in patients with low MGMT expression or hypermethylated MGMT promoters may reprogram the chemotherapeutic sensitivity of TMZ-R tumors.

## Supplementary information

Supplementary information

Original western blots

## Data Availability

The data that were analyzed during the current study are available from the corresponding author on reasonable request.

## References

[1] Alifieris C, Trafalis DT (2015). Glioblastoma multiforme: pathogenesis and treatment. Pharmacol Ther.

[2] Stupp R, Mason WP, van den Bent MJ, Weller M, Fisher B, Taphoorn MJ (2005). Radiotherapy plus concomitant and adjuvant temozolomide for glioblastoma. N Engl J Med.

[3] Baumann F, Bjeljac M, Kollias SS, Baumert BG, Brandner S, Rousson V (2004). Combined thalidomide and temozolomide treatment in patients with glioblastoma multiforme. J Neuro-Oncol.

[4] Esteller M, Garcia-Foncillas J, Andion E, Goodman SN, Hidalgo OF, Vanaclocha V (2000). Inactivation of the DNA-repair gene MGMT and the clinical response of gliomas to alkylating agents. N Engl J Med.

[5] Hirose Y, Kreklau EL, Erickson LC, Berger MS, Pieper RO (2003). Delayed repletion of O6-methylguanine-DNA methyltransferase resulting in failure to protect the human glioblastoma cell line SF767 from temozolomide-induced cytotoxicity. J Neurosurg.

[6] Brandes AA, Franceschi E, Paccapelo A, Tallini G, De Biase D, Ghimenton C (2017). Role of MGMT methylation status at time of diagnosis and recurrence for patients with glioblastoma: clinical implications. Oncologist.

[7] Hegi ME, Diserens AC, Gorlia T, Hamou MF, de Tribolet N, Weller M (2005). MGMT gene silencing and benefit from temozolomide in glioblastoma. N Engl J Med.

[8] Brennan CW, Verhaak RG, McKenna A, Campos B, Noushmehr H, Salama SR (2013). The somatic genomic landscape of glioblastoma. Cell.

[9] Happold C, Roth P, Wick W, Schmidt N, Florea AM, Silginer M (2012). Distinct molecular mechanisms of acquired resistance to temozolomide in glioblastoma cells. J Neurochem.

[10] Yi GZ, Liu YW, Xiang W, Wang H, Chen ZY, Xie SD (2016). Akt and beta-catenin contribute to TMZ resistance and EMT of MGMT negative malignant glioma cell line. J Neurol Sci.

[11] Gil Del Alcazar CR, Todorova PK, Habib AA, Mukherjee B, Burma S (2016). Augmented HR repair mediates acquired temozolomide resistance in glioblastoma. Mol cancer Res: MCR.

[12] An Z, Weiss WA (2016). Cholesterol: an achilles’ heel for glioblastoma?. Cancer Cell.

[13] Kitange GJ, Mladek AC, Carlson BL, Schroeder MA, Pokorny JL, Cen L (2012). Inhibition of histone deacetylation potentiates the evolution of acquired temozolomide resistance linked to MGMT upregulation in glioblastoma xenografts. Clin Cancer Res.

[14] Jiao J, Zhang R, Li Z, Yin Y, Fang X, Ding X (2018). Nuclear Smad6 promotes gliomagenesis by negatively regulating PIAS3-mediated STAT3 inhibition. Nat Commun.

[15] Asp M, Giacomello S, Larsson L, Wu C, Furth D, Qian X (2019). A spatiotemporal organ-wide gene expression and cell atlas of the developing human heart. Cell.

[16] Qiu X, Hill A, Packer J, Lin D, Ma YA, Trapnell C (2017). Single-cell mRNA quantification and differential analysis with Census. Nat Methods.

[17] Hanzelmann S, Castelo R, Guinney J (2013). GSVA: gene set variation analysis for microarray and RNA-seq data. BMC Bioinform.

[18] Yeong J, Suteja L, Simoni Y, Lau KW, Tan AC, Li HH (2021). Intratumoral CD39(+)CD8(+) T cells predict response to programmed cell death protein-1 or programmed death ligand-1 blockade in patients with NSCLC. J Thorac Oncol.

[19] Yeong J, Tan T, Chow ZL, Cheng Q, Lee B, Seet A (2020). Multiplex immunohistochemistry/immunofluorescence (mIHC/IF) for PD-L1 testing in triple-negative breast cancer: a translational assay compared with conventional IHC. J Clin Pathol.

[20] Yin Y, Zhang X, Li Z, Deng L, Jiao G, Zhang B (2013). Glucocorticoid receptor beta regulates injury-mediated astrocyte activation and contributes to glioma pathogenesis via modulation of beta-catenin/TCF transcriptional activity. Neurobiol Dis.

[21] Silva R, Vilas-Boas V, Carmo H, Dinis-Oliveira RJ, Carvalho F, de Lourdes Bastos M (2015). Modulation of P-glycoprotein efflux pump: induction and activation as a therapeutic strategy. Pharmacol therapeutics.

[22] Robey RW, Pluchino KM, Hall MD, Fojo AT, Bates SE, Gottesman MM (2018). Revisiting the role of ABC transporters in multidrug-resistant cancer. Nat Rev Cancer.

[23] Gourbal B, Sonuc N, Bhattacharjee H, Legare D, Sundar S, Ouellette M (2004). Drug uptake and modulation of drug resistance in Leishmania by an aquaglyceroporin. J Biol Chem.

[24] Yi GZ, Huang G, Guo M, Zhang X, Wang H, Deng S (2019). Acquired temozolomide resistance in MGMT-deficient glioblastoma cells is associated with regulation of DNA repair by DHC2. Brain J Neurol.

[25] Colak S, Ten Dijke P (2017). Targeting TGF-beta signaling in cancer. Trends Cancer.

[26] Alimbetov D, Askarova S, Umbayev B, Davis T, Kipling D (2018). Pharmacological targeting of cell cycle, apoptotic and cell adhesion signaling pathways implicated in chemoresistance of cancer cells. Int J Mol Sci.

[27] Guo F, Zhang H, Jia Z, Cui M, Tian J (2018). Chemoresistance and targeting of growth factors/cytokines signalling pathways: towards the development of effective therapeutic strategy for endometrial cancer. Am J Cancer Res.

[28] Yang L, Shi P, Zhao G, Xu J, Peng W, Zhang J (2020). Targeting cancer stem cell pathways for cancer therapy. Signal Transduct Target Ther.

[29] Mukherjee S (2020). Quiescent stem cell marker genes in glioma gene networks are sufficient to distinguish between normal and glioblastoma (GBM) samples. Sci Rep.

[30] Banik A, Sharma R, Chauhan A, Singh S (2022). Cutting the umbilical cord: cancer stem cell-targeted therapeutics. Life Sci.

[31] Sun X, Kaufman PD (2018). Ki-67: more than a proliferation marker. Chromosoma..

[32] Zheng GX, Terry JM, Belgrader P, Ryvkin P, Bent ZW, Wilson R (2017). Massively parallel digital transcriptional profiling of single cells. Nat Commun.

[33] Rutka JT, Ivanchuk S, Mondal S, Taylor M, Sakai K, Dirks P (1999). Co-expression of nestin and vimentin intermediate filaments in invasive human astrocytoma cells. Int J Dev Neurosci.

[34] Sigismund S, Avanzato D, Lanzetti L (2018). Emerging functions of the EGFR in cancer. Mol Oncol.

[35] Cho DY, Lin SZ, Yang WK, Lee HC, Hsu DM, Lin HL (2013). Targeting cancer stem cells for treatment of glioblastoma multiforme. Cell Transpl.

[36] Stepanenko AA, Chekhonin VP. On the critical issues in temozolomide research in glioblastoma: clinically relevant concentrations and MGMT-independent resistance. Biomedicines. 2019;7. 10.3390/biomedicines7040092 PMC6966644 31783653

[37] Rich JN, Bao S (2007). Chemotherapy and cancer stem cells. cell stem cell.

[38] Olmez I, Shen W, McDonald H, Ozpolat B (2015). Dedifferentiation of patient-derived glioblastoma multiforme cell lines results in a cancer stem cell-like state with mitogen-independent growth. J Cell Mol Med.

[39] Tejero R, Huang Y, Katsyv I, Kluge M, Lin JY, Tome-Garcia J (2019). Gene signatures of quiescent glioblastoma cells reveal mesenchymal shift and interactions with niche microenvironment. EBioMedicine.

[40] Lawlor K, Marques-Torrejon MA, Dharmalingham G, El-Azhar Y, Schneider MD, Pollard SM (2020). Glioblastoma stem cells induce quiescence in surrounding neural stem cells via Notch signaling. Genes Dev.

[41] Sachdeva R, Wu M, Johnson K, Kim H, Celebre A, Shahzad U (2019). BMP signaling mediates glioma stem cell quiescence and confers treatment resistance in glioblastoma. Sci Rep.

[42] Garnier D, Meehan B, Kislinger T, Daniel P, Sinha A, Abdulkarim B (2018). Divergent evolution of temozolomide resistance in glioblastoma stem cells is reflected in extracellular vesicles and coupled with radiosensitization. Neuro Oncol.

[43] Chan KS (2016). Molecular pathways: targeting cancer stem cells awakened by chemotherapy to abrogate tumor repopulation. Clin Cancer Res.

[44] Nunes T, Hamdan D, Leboeuf C, El Bouchtaoui M, Gapihan G, Nguyen TT (2018). Targeting cancer stem cells to overcome chemoresistance. Int J Mol Sci.

[45] Jinushi M, Chiba S, Yoshiyama H, Masutomi K, Kinoshita I, Dosaka-Akita H (2011). Tumor-associated macrophages regulate tumorigenicity and anticancer drug responses of cancer stem/initiating cells. Proc Natl Acad Sci USA.

[46] Tang YA, Chen YF, Bao Y, Mahara S, Yatim S, Oguz G (2018). Hypoxic tumor microenvironment activates GLI2 via HIF-1alpha and TGF-beta2 to promote chemoresistance in colorectal cancer. Proc Natl Acad Sci USA.

[47] Singh N, Miner A, Hennis L, Mittal S (2021). Mechanisms of temozolomide resistance in glioblastoma—a comprehensive review. Cancer Drug Resist.

[48] Cai Q, Zhu A, Gong L (2018). Exosomes of glioma cells deliver miR-148a to promote proliferation and metastasis of glioblastoma via targeting CADM1. Bull Cancer.

[49] Li C, Wang Y, Wang H, Wang B, Li N, Qin Y (2021). miR-486 promotes the invasion and cell cycle progression of ovarian cancer cells by targeting CADM1. Anal Cell Pathol.

[50] Zhang W, Xie HY, Ding SM, Xing CY, Chen A, Lai MC (2016). CADM1 regulates the G1/S transition and represses tumorigenicity through the Rb-E2F pathway in hepatocellular carcinoma. Hepatobiliary Pancreat Dis Int.

[51] Areeb Z, Stuart SF, West AJ, Gomez J, Nguyen HPT, Paradiso L (2020). Reduced EGFR and increased miR-221 is associated with increased resistance to temozolomide and radiotherapy in glioblastoma. Sci Rep.

[52] Jun HJ, Bronson RT, Charest A (2014). Inhibition of EGFR induces a c-MET-driven stem cell population in glioblastoma. Stem Cells.

[53] Marques-Torrejon MA, Williams CAC, Southgate B, Alfazema N, Clements MP, Garcia-Diaz C (2021). LRIG1 is a gatekeeper to exit from quiescence in adult neural stem cells. Nat Commun.

[54] Liu B, Guo Z, Dong H, Daofeng T, Cai Q, Ji B (2015). LRIG1, human EGFR inhibitor, reverses multidrug resistance through modulation of ABCB1 and ABCG2. Brain Res.

[55] Gao X, Xia X, Li F, Zhang M, Zhou H, Wu X (2021). Circular RNA-encoded oncogenic E-cadherin variant promotes glioblastoma tumorigenicity through activation of EGFR-STAT3 signalling. Nat Cell Biol.

[56] Kohsaka S, Wang L, Yachi K, Mahabir R, Narita T, Itoh T (2012). STAT3 inhibition overcomes temozolomide resistance in glioblastoma by downregulating MGMT expression. Mol Cancer Ther.

[57] Yang PL, Liu LX, Li EM, Xu LY (2020). STAT3, the challenge for chemotherapeutic and radiotherapeutic efficacy. Cancers.

[58] Struve N, Binder ZA, Stead LF, Brend T, Bagley SJ, Faulkner C (2020). EGFRvIII upregulates DNA mismatch repair resulting in increased temozolomide sensitivity of MGMT promoter methylated glioblastoma. Oncogene..

[59] Li L, Huang Y, Gao Y, Shi T, Xu Y, Li H (2018). EGF/EGFR upregulates and cooperates with Netrin-4 to protect glioblastoma cells from DNA damage-induced senescence. BMC Cancer.

[60] Nitta Y, Shimizu S, Shishido-Hara Y, Suzuki K, Shiokawa Y, Nagane M (2016). Nimotuzumab enhances temozolomide-induced growth suppression of glioma cells expressing mutant EGFR in vivo. Cancer Med.

[61] Grogan PT, Sarkaria JN, Timmermann BN, Cohen MS (2014). Oxidative cytotoxic agent withaferin A resensitizes temozolomide-resistant glioblastomas via MGMT depletion and induces apoptosis through Akt/mTOR pathway inhibitory modulation. Investig N Drugs.

[62] Lenting K, Verhaak R, Ter Laan M, Wesseling P, Leenders W (2017). Glioma: experimental models and reality. Acta Neuropathol.

[63] Vidal SJ, Rodriguez-Bravo V, Galsky M, Cordon-Cardo C, Domingo-Domenech J (2014). Targeting cancer stem cells to suppress acquired chemotherapy resistance. Oncogene..

