# Supplementary material for: Characterization of EGFR-reprogrammable temozolomide-resistant cells in a model of glioblastoma
Source: Cell Death Discov. 2022 Oct 31;8:438. doi: 10.1038/s41420-022-01230-y (PMC9622861; doi:10.1038/s41420-022-01230-y)
Supplement: Supplementary file 1 — Supplementary information [file 41420_2022_1230_MOESM1_ESM.docx]

**
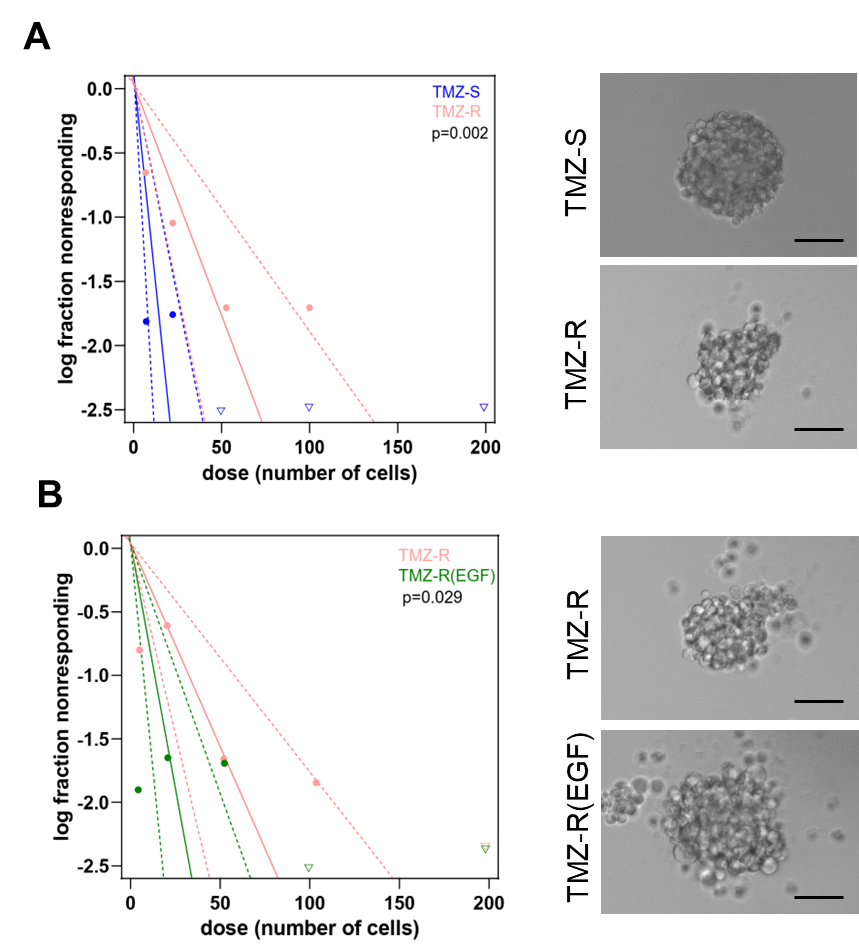
**

**Supplementary Figure 1. Limiting dilution assay (LDA) detecting the cancer stem cells (CSCs) sphere formation efficiency.**

**A.** LDA analysis of TMZ-S cells and TMZ-R cells. Representative images of spheres derived from 50 cells.

**B.** LDA analysis of TMZ-R cells and TMZ-R cells in culture medium with EGF. Representative images of spheres derived from 50 cells. Bars, 200 µm.


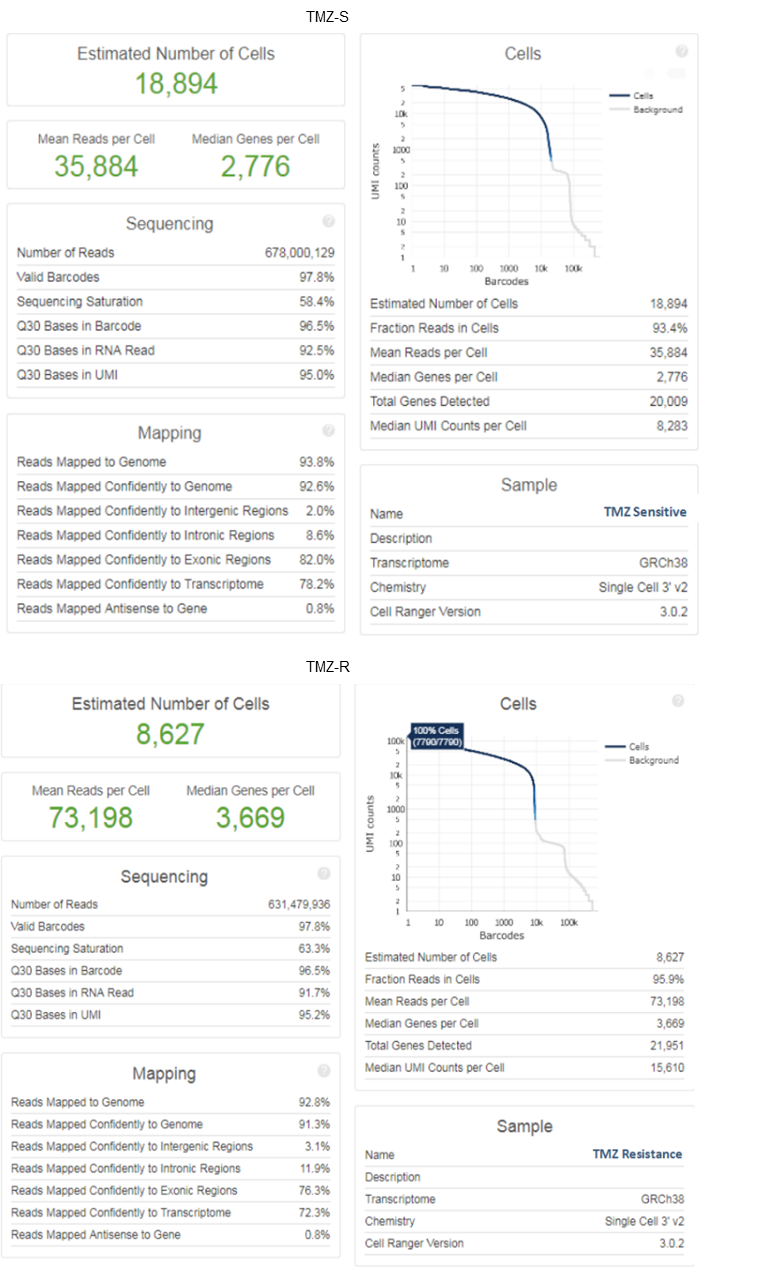
**Supplementary Figure 2. Overview of scRNA-seq analysis of cells derived from TMZ-S and TMZ-R tumor.**

Microfluidicbased approaches of the 10 x Genomics® platform was performed to obtain the transcriptome of cells isolated from TMZ-S and TMZ-R tissues. The estimated cell number, the reads and the mean genes per cell were indicated.


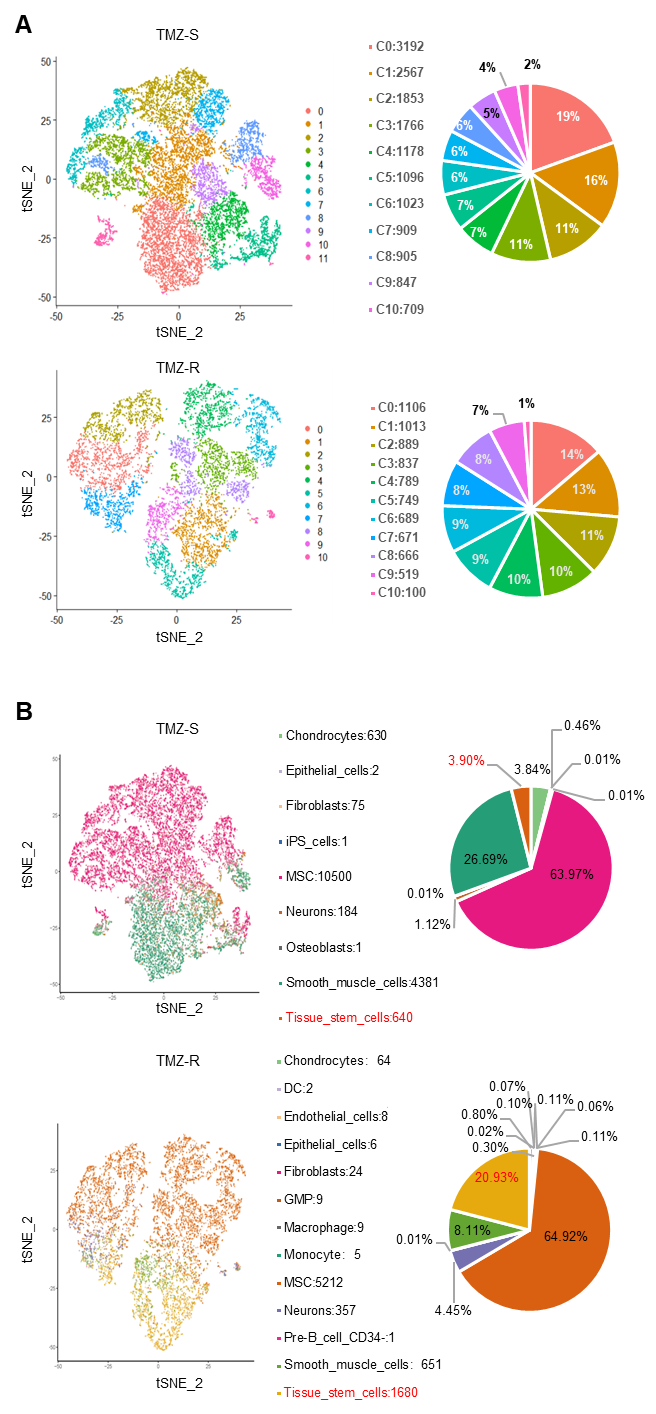


**Supplementary Figure 3. TMZ-R tumors contain a higher proportion of stem cell populations.**

**A.** Graph-Based clustering identified 12 distinct cell clusters in TMZ-S tumor cells and 11 in TMZ-R tumor via t-distributed stochastic neighbor embedding (t-SNE).

**B.** SingleR annotation indicating a marked increase of tissue stem cell population in TMZ-R as compared with it in TMZ-S.


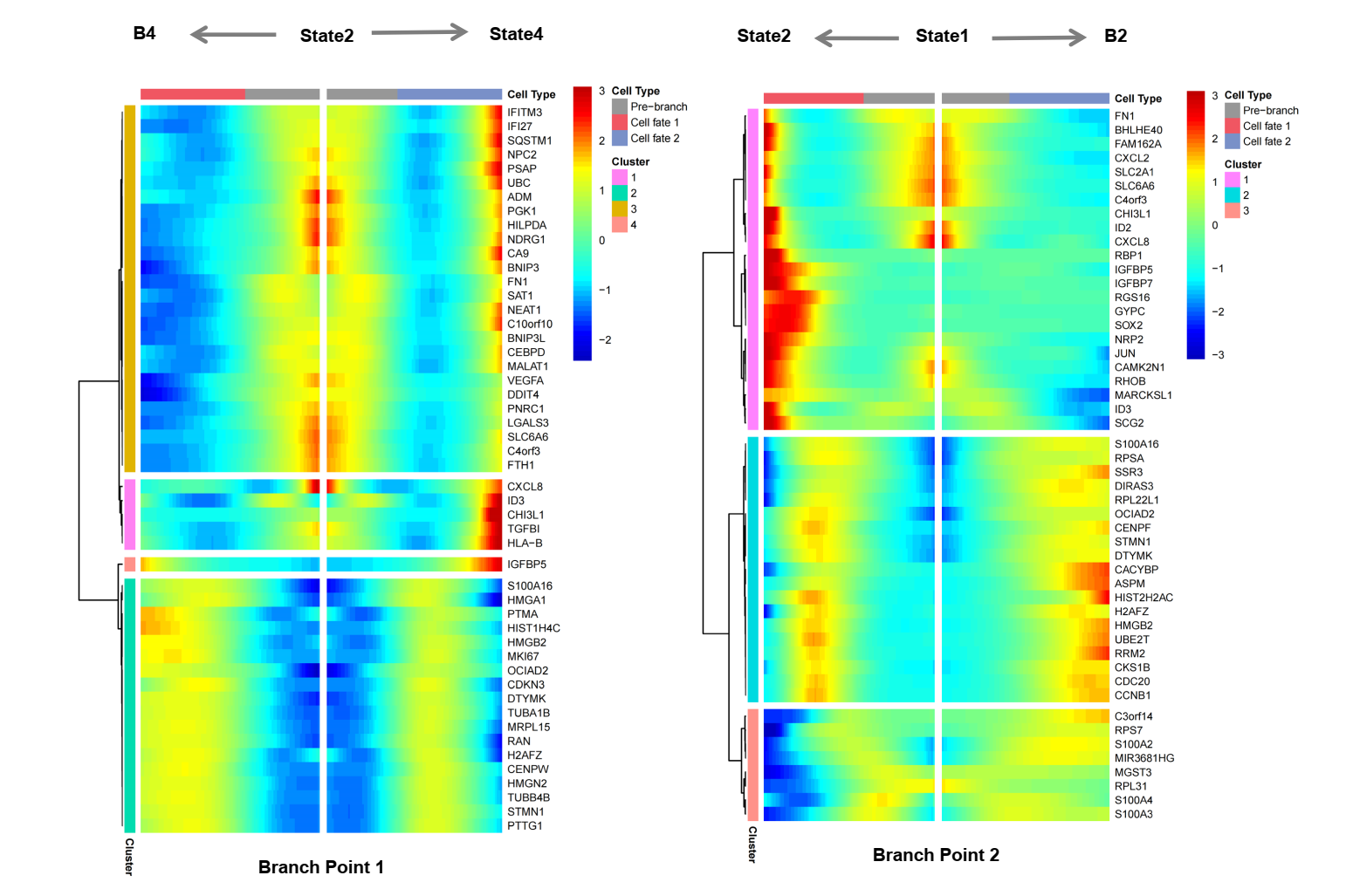


**Supplementary Figure 4. Heatmap showing the top 50 genes in TMZ-R with branch-dependent expression (qval < 1e-4) for branch point 2 and 1.**


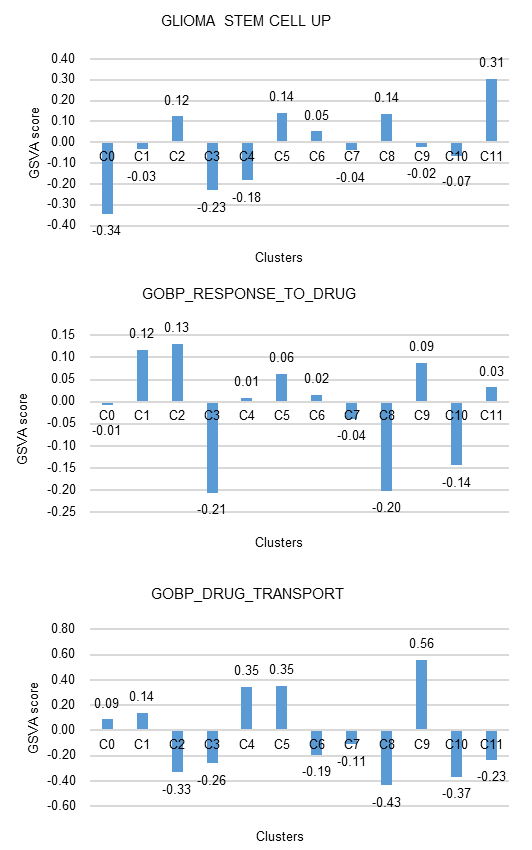


**Supplementary Figure 5. GSVA analysis displaying cellular behavior difference among cell clusters in TMZ-S. Bar charts visualizing GSVA scores of indicated genesets in GO-BP terms.**


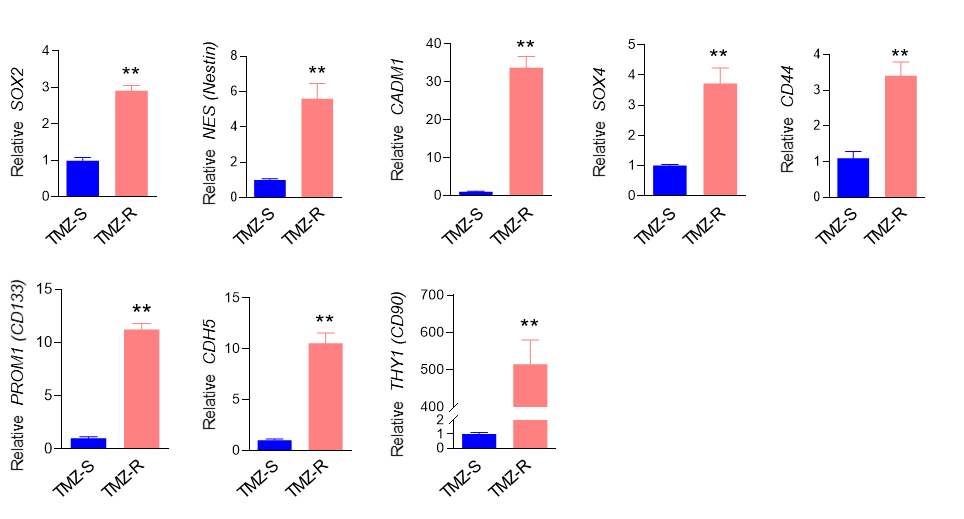


**Supplementary Figure 6. qRT–PCR assay comparing the expression of indicated mRNA in between TMZ-S and TMZ-R tumors (n=3, **P<0.01)**


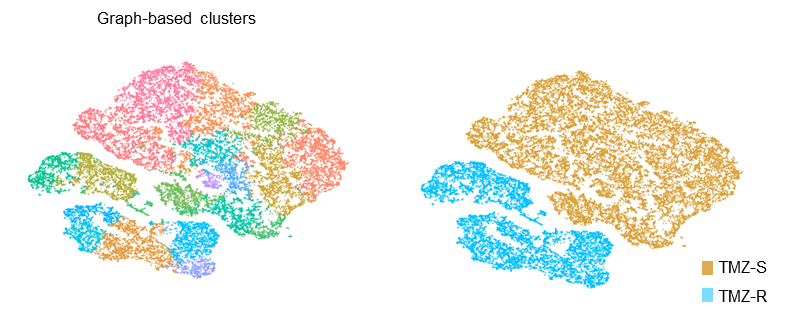


**Supplementary Figure 7. Graph-based clustering identifying 20 different subgroups based on cluster-specific genes and biological processes derived from total 27,521 cells covering TMZ-R and TMZ-S libraries (left), and indicating the distribution of cells derived from TMZ-S and TMZ-R (right).**

**Supplementary Table 1. Primers for qPCR**

| **Gene** | **Forward** | **Reverse** |
| --- | --- | --- |
| *ABCB1* | TTGCTGCTTACATTCAGGTTTCA | AGCCTATCTCCTGTCGCATTA |
| *ABCC3* | TGGGGTGAAGTTTCGTACTGG | CACGTTTGACTGAGTTGGTGATA |
| *SOX2* | GCTACAGCATGATGCAGGACCA | TCTGCGAGCTGGTCATGGAGTT |
| *NES* | TCAAGATGTCCCTCAGCCTGGA | AAGCTGAGGGAAGTCTTGGAGC |
| *CADM1* | ATGGCGAGTGTAGTGCTGC | GATCACTGTCACGTCTTTCGT |
| *SOX4* | GAAATGACCCGAGAACCC | CTCCCCAACGTGCAAAT |
| *CD44* | CCAGAAGGAACAGTGGTTTGGC | ACTGTCCTCTGGGCTTGGTGTT |
| *PROM1* | CACTACCAAGGACAAGGCGTTC | CAACGCCTCTTTGGTCTCCTTG |
| *CDH5* | AAGCGTGAGTCGCAAGAATG | TCTCCAGGTTTTCGCCAGTG |
| *THY1* | TCACCCATCCAGTACGAGTTC | GGAGCGGTATGTGTGCTCAG |
| *GAPDH* | TGTTGCCATCAATGACCCCTT | CTCCACGACGTACTCAGCG |

**Supplementary Table 2. Primers for semi-quantitative PCR**

| **Gene** | **Product Size** | **Forward** | **Reverse** |
| --- | --- | --- | --- |
| *MGMT* | 157 bp | TTTTCCAGCAAGAGTCGTTCAC | GGGACAGGATTGCCTCTCAT |
| *GAPDH* | 658 bp | ATCACCATCTTCCAGGAGC | AGGAGTGGGTGTCGCTGT |
| *MGMT(M)* | 159bp | CGTAGTCGTTTCGAGTAGGATC | ACCTTAATTTACCAAATAACCCGTA |
| *MGMT(U)* | 160bp | GTGTAGTTGTTTTGAGTAGGATTGGACCTT | ACCTTAATTTACCAAATAACCCATA |

**Supplementary Table 3. List of antibodies**

| **Antigen** | **Primary Antibody** | **Dilution** |
| --- | --- | --- |
| Nestin | Sigma; HPA046113; mouse monoclonal | 1:5000 for WB; 1:200 for IF  1:500 for IHC |
| SOX2 | ABCAM; AB97959; rabbit polyclonal | 1:1000 for WB; 1:600 for IF  1:500 for IHC |
| CADM1 | ABCAM; AB216585; Rabbit polyclonal | 1:400 for WB; 1:500 for IHC |
| PE-CD133 | Miltenyi Biotec; 130-111-80; mouse monoclonal | 1:5 for FC |
| FITC-CD44 | BD; 555478; mouse monoclonal | 1:5 for FC |
| STAT3 | Cell Signaling; 9139; mouse monoclonal | 1:1000 for WB |
| Phospho-STAT3 (Tyr705) | Cell Signaling; 9145; rabbit monoclonal | 1:1000 for WB |
| EGFR | Cell Signaling; 4267; rabbit monoclonal | 1:1000 for WB; 1:200 for IF |
| EGFR | ThermoFisher; MA5-13070; mouse monoclonal | 1:5000 for WB; 1:400 for IF  1:500 for IHC |
| Phospho-EGFR (Tyr1068) | Cell Signaling; 3777; rabbit monoclonal | 1:1000 for WB |
| Akt | Cell Signaling; 4691; rabbit monoclonal | 1:1000 for WB |
| Phospho-Akt (Ser473) | Cell Signaling; 4060; rabbit monoclonal | 1:1000 for WB |
| Normal Rabbit IgG | Cell Signaling; 2729 | 1:100 for IP or ChIP; 1:500 for IHC or IF |
| Normal Mouse IgG | ABCAM; ab188776 | 1:100 for IP; 1:500 for IF |
| GAPDH | ThermoFisher; MA5-15738-1MG; mouse monoclonal | 1:2000 for WB |
| β-actin | ThermoFisher; MA5-15738-1MG; mouse monoclonal | 1:5000 for WB |
| Ki67 | Proteintech 27309-1-AP; mouse monoclonal | 1:5000 for WB; 1:800 for IF |

| **Supplementary Table 4. Markers of cancer stem cell in human brain** | | | | |
| --- | --- | --- | --- | --- |
| **Marker** | **Supported NO.** | **Gene symbol** | **Entrez ID** | **David Gene Name** |
| ALDH1 | 1 | ALDH1A1 | 216 | aldehyde dehydrogenase 1 family member A1(ALDH1A1) |
| ASCL1 | 1 | ASCL1 | 429 | achaete-scute family bHLH transcription factor 1(ASCL1) |
| BMI1 | 1 | BMI1 | 648 | BMI1 proto-oncogene, polycomb ring finger (BMI1) |
| BOC | 1 | BOC | 91653 | BOC cell adhesion associated, oncogene regulated (BOC) |
| CADM1 | 1 | CADM1 | 23705 | cell adhesion molecule 1(CADM1) |
| CCND2 | 1 | CCND2 | 894 | cyclin D2(CCND2) |
| CD24 | 1 | CD24 | 100133941 | CD24 molecule (CD24) |
| CD44 | 1 | CD44 | 960 | CD44 molecule (Indian blood group) (CD44) |
| CDH5 | 1 | CDH5 | 1003 | cadherin 5(CDH5) |
| CHD7 | 1 | CHD7 | 55636 | chromodomain helicase DNA binding protein 7(CHD7) |
| CLCC1 | 1 | CLCC1 | 23155 | chloride channel CLIC like 1(CLCC1) |
| β-catenin | 1 | CTNNB1 | 1499 | catenin beta 1(CTNNB1) |
| EGFR | 1 | EGFR | 1956 | epidermal growth factor receptor (EGFR) |
| CD15 | 3 | FUT4 | 2526 | fucosyltransferase 4(FUT4) |
| GD2 | 1 | GBA | 2629 | glucosylceramidase beta (GBA) |
| HMOX1 | 1 | HMOX1 | 3162 | heme oxygenase 1(HMOX1) |
| HOXD9 | 1 | HOXD9 | 3235 | homeobox D9(HOXD9) |
| Musashi-1 | 2 | MSI1 | 4440 | musashi RNA binding protein 1(MSI1) |
| NANOG | 1 | NANOG | 79923 | Nanog homeobox (NANOG) |
| Nestin | 9 | NES | 10763 | Nestin (NES) |
| NFIB | 1 | NFIB | 4781 | nuclear factor I B(NFIB) |
| CD271 | 1 | NGFR | 4804 | nerve growth factor receptor (NGFR) |
| OCT4 | 1 | POU5F1 | 5460 | POU class 5 homeobox 1(POU5F1) |
| CD133 | 23 | PROM1 | 8842 | prominin 1(PROM1) |
| SCAMP3 | 1 | SCAMP3 | 10067 | secretory carrier membrane protein 3 (SCAMP3) |
| SLC16A1 | 1 | SLC16A1 | 6566 | solute carrier family 16 member 1 (SLC16A1) |
| GLUT3 | 1 | SLC2A3 | 6515 | solute carrier family 2 member 3 (SLC2A3) |
| SOX11 | 1 | SOX11 | 6664 | SRY-box 11(SOX11) |
| SOX2 | 8 | SOX2 | 6657 | SRY-box 2(SOX2) |
| SOX4 | 1 | SOX4 | 6659 | SRY-box 4(SOX4) |
| TCF4 | 1 | TCF4 | 6925 | transcription factor 4(TCF4) |
| CD90 | 2 | THY1 | 7070 | Thy-1 cell surface antigen (THY1) |
