# Supplementary figures and images for: Characterization of EGFR-reprogrammable temozolomide-resistant cells in a model of glioblastoma
Source: Cell Death Discov. 2022 Oct 31;8:438. doi: 10.1038/s41420-022-01230-y (PMC9622861; doi:10.1038/s41420-022-01230-y)

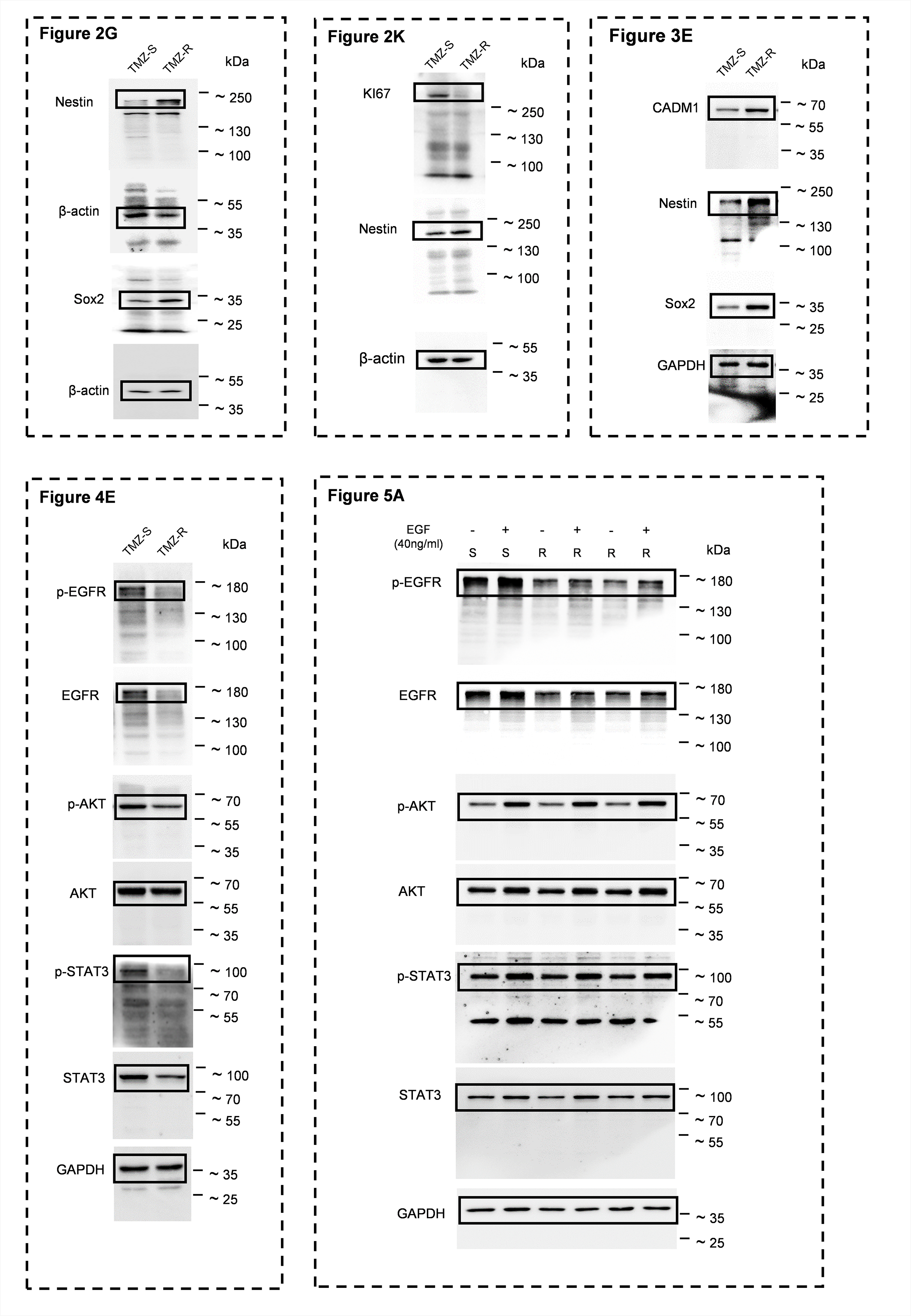

Supplement: Supplementary file 2 — Original western blots [file 41420_2022_1230_MOESM2_ESM.tif]
